# Insights into the genetic determination of tuber shape and eye depth in potato natural population based on autotetraploid potato genome

**DOI:** 10.3389/fpls.2023.1080666

**Published:** 2023-03-28

**Authors:** Long Zhao, Meiling Zou, Ke Deng, Chengcai Xia, Sirong Jiang, Chenji Zhang, Yongzhen Ma, Xiaorui Dong, Miaohua He, Tiancang Na, Jian Wang, Zhiqiang Xia, Fang Wang

**Affiliations:** ^1^Academy of Agriculture and Forestry Sciences, Qinghai University, Xining, China; ^2^National Key Laboratory of Sanjiangyuan Ecology and Plateau Agriculture and Animal Husbandry, Qinghai University, Xining, China; ^3^College of Tropical Crops, Sanya Nanfan Research Institute, Hainan University, Hainan Yazhou Bay Seed Laboratory, Sanya, China; ^4^College of Agronomy and Biotechnology, China Agricultural University, Beijing, China; ^5^Key Laboratory of Qinghai-Tibet Plateau Biotechnology Ministry of Education, Qinghai University, Xining, China; ^6^Qinghai Provincial Key Laboratory of Potato Breeding, Qinghai University, Xining, China; ^7^Laboratory for Research and Utilization of Qinghai Tibet Plateau Germplasm Resources, Qinghai University, Xining, China

**Keywords:** tetraploid potato, GWAS, tuber shape, eye depth, population structure, genetic diversity

## Abstract

Potato is one of the world’s most important food crops, with a time-consuming breeding process. In this study, we performed a genome-wide association (GWAS) analysis of the two important traits of potato tuber shape and eye depth, using the tetraploid potato genome (2n=4x=48) as a reference. A total of 370 potatoes were divided into three subgroups based on the principal component analysis and evolutionary tree analysis. The genetic diversity within subgroups is low (5.18×10^-5^, 4.36×10^-5^ and 4.24×10^-5^). Genome-wide linkage disequilibrium (LD) analysis showed that their LD is about 60 Kb. GWAS analysis identified that 146 significant single nucleotide polymorphism (SNP) loci at Chr01A1:34.44−35.25 Mb and Chr02A1:28.35−28.54 Mb regions are significantly associated with potato tuber shape, and that three candidate genes that might be related to potato tuber traits, *PLATZ* transcription factor, *UTP-glucose-1-phosphate uridylyltransferase* and *FAR1* DNA-binding domain, are in the association region of Chr02A1. GWAS analysis identified 53 significant SNP loci at Chr05A2: 49.644-50.146 Mb and Chr06A2: 25.866-26.384 Mb regions with robust associations with potato tuber eye depth. Hydrolase and methyltransferases are present in the association region of Chr05A2, and three CYPs are present in the association region of Chr06A2. Our findings suggested that these genes are closely associated with potato tuber shape and eye depth. Our study identified molecular markers and candidate genes for improving tetraploid potato tuber shape and eye depth and provided ideas and insights for tetraploid potato breeding.

## Introduction

Potato (*Solanum tuberosum* L.) is native to the Andean region of South America and has been cultivated in southern Peruvian provinces dating back to 8,000 to 5,000 years ago. Potato contains a large amount of starch, protein and trace elements the human body needs. It is one of the most important food crops in the world and ranks third in the global total output after wheat and rice in the world (Birch et al., 2012). China is the largest potato producer in the world and potato has become the fourth largest staple food in China. As the fourth largest staple food of 1.3 billion people in China, potato cultivation is crucial to ensuring China’s food security, precise poverty alleviation and industrial chain structure adjustment. With global potato production growing gradually, many countries have become dependent on potatoes for food production (Stokstad, 2019) and the planting area in developing countries has surpassed that in developed countries. The major potato producers include China, Russia, India, Ukraine and the United States. Except for South America, the genetic base of cultivated potatoes is narrow. Unlike other crops, potatoes have been slow to progress toward inbreeding, and the genetic complexity of commercial tetraploid cultivars is a key factor impeding the genetic improvement of cultivated potatoes (Zhang et al., 2021).

Tuber shape is an important agronomic trait for identifying potato varieties and one of the most important quality traits in the appearance of potato chips or fries. Potato tuber shape is determined by the cell division and enlargement rate in all directions during tuber formation. Selecting and breeding potatoes with different shapes is essential to meet the requirements of people with different consumption habits, accelerate the development of the potato processing industry, and promote economic growth. Jong and Rowe (1972) and Taylor (1978) studied diploid and tetraploid potato materials, respectively, and revealed a master effect gene controlling potato tuber shape, in which round shape predominated over elongated shape (Jong and Rowe, 1972; Taylor, 1978) Subsequently, Van Eck et al. (1994) mapped the gene on chromosome 10 using a small diploid F1 population with 50 genotypes (Van Eck et al., 1994). Other related reports indicated that three genes on chromosomes1, 2, 4 and 10 are crucial genetic motifs for round versus elongated potato shape (Hara-Skrzypiec et al., 2018). Researchers found that potato tuber shape is a continuously distributed phenotype based on the ratio of tuber length to tuber width and identified a quantitative trait locus (QTL) on the site mapping of chromosome 10. Further fine mapping of tuber shape genes based on a diploid segregating population led to the construction of a high-density genetic linkage map of the *Ro* region on chromosome 10 (Lindqvist-Kreuze et al., 2015; Fan et al., 2022). Besides the tuber shape, tuber eye depth is also an important trait determining potato varieties’ appearance, quality, and processing suitability. A deeper eye will increase the volume loss during peeling and the cost of potato processing. Previous studies showed that tuber eye depth QTLs are mainly on chromosomes 2 and 10 (Li et al., 2005; Prashar et al., 2014). However, it was also shown that chromosomes 1, 3, 4, 5 and 11 have QTL loci and they are related to tuber eye depth (Hara-Skrzypiec et al., 2018).

Following the development of sequencing technology and advances in genomics research, re-sequencing-based GWAS have become essential for studying complex traits in rice (Huang et al., 2010; Huang et al., 2011), foxtail millet (Jia et al., 2013), maize (Kump et al., 2011), Arabidopsis (Atwell et al., 2010), soybean (Zhou et al., 2015), tomato (Lin et al., 2014) and sorghum (Morris et al., 2013). The combination of population structure and GWAS provides a new way of localizing key genes. The combination of population structure and GWAS provides a new way of localizing key genetic genes. At present, the reported studies of potato GWAS are mainly based on haploid reference genome, and few studies of GWAS use tetraploid potato reference genome. Wang et al. conducted a pilot GWAS of late blight potatoes and mined 14 candidate genes (Wang et al., 2021). However, the study was limited to the reference genome version, which was selected from diploid DM potato varieties. High-quality reference genomes are important in studies on crop improvement, effective tracking of genomic variations, localization of important QTLs, and discovery of new alleles (Morrell et al., 2011; Xie et al., 2019). With the development of sequencing technology, the genomes of different potato varieties and ploidy were gradually revealed, especially the assembly of the genomes of tetraploid cultivated potatoes Otava (Sun et al., 2022), Atlantic and Castle Russet (Hoopes et al., 2022), C88 (Bao et al., 2022) and Q9 (Wang et al., 2022) which has laid a foundation for understanding the genetic mechanism of tetraploid potato and is of great significance in breeding common tetraploid cultivated potato varieties.

Previous studies on the agronomic traits of tetraploid potato have mainly used the diploid potato DM (The Potato Genome Sequencing Consortium, 2011) as the reference genome. However, few studies of GWAS using the tetraploid genome as a reference (2n=4x=48) and on agronomic traits in large-scale natural populations are reported. In this study, we collected 370 tetraploid potatoes from all over the world and conducted a GWAS to explore the population structure and genetic diversity of germplasm resources with the genome of tetraploid cultivated potato Q9 (Wang et al., 2022) as the reference. In addition, we performed GWAS analysis to identify highly phenotype-related loci and shape-related candidate genes. Our results provide a reference for breeding tetraploid potato varieties with shallow tuber eye depth and desired tuber shape and variant loci and gene resources to further study the underlying genetic mechanisms.

## Materials and methods

### Plant materials and phenotyping

The 370 tetraploid potato varieties used in this study were collected from around the world and grown at the Qinghai Plateau Potato Experiment Station (36°680N, 101°260E), College of Agriculture and Forestry, Qinghai University. Their tuber shape and eye depth data were collected from 2020−2021 and used for GWAS analysis. Since the traits of tuber shape and eye depth are mainly quality traits and have a relatively stable performance, the data were collected in our study for only two years. We measured the shape index (size of length/size of width), according to the national standards of the People’s Republic of China. The tuber shape was expressed in grades 1-6, with grade 1 for nearly round tuber shapes and grade 6 for extremely long tubers (**Table 1**). We selected potato tubers with a width of about 3 cm for tuber eye depth identification, and compared them with standard cultivar to determine their grade. The tuber eye depth was expressed in grades 1-9, with grade 1 for the extremely shallow and grade 9 for the extremely depth (**Table 1**). Traits were identified according to the national standards of the People’s Republic of China (GB/T 19557.28-2018; https://openstd.samr.gov.cn/bzgk/gb/). A randomized complete block design with three repetitions were used to grow the 370 cultivars.

**Table 1:** Potato tuber shape and eye depth identification standards.

Agronomic Trait		The Name of Standard Cultivar	Index
Tuber shape	Round	Kexin 12	1
	Short oval	Kexin 4	2
	Oval	Dongnong 303	3
	Oblong	Zhongshu 9	4
	Long shape	Spunta	5
	Extremely shape	Russet Burbank	6
Tuber eye depth	Extremely shallow	–	1
	Extremely shallow to shallow	–	2
	shallow	Zhongshu 3	3
	Shallow to Medium	–	4
	Medium	Kexin 4	5
	Medium to deep	–	6
	Deep	Hua 525	7
	Deep to extremely deep	–	8
	Extremely deep	–	9

### DNA extraction and sequencing

Potato DNA samples were extracted using a plant DNA extraction kit (QIAGEN). DNA quality was measured by OD_260nm_/OD_280nm_ ratio using a spectrophotometer and by agarose gel electrophoresis for RNA or protein contaminations. DNA concentration was quantified using Qubit. The qualified genomic DNA was fragmented to 300-350 bp using an ultrasonic interrupter and purified using magnetic beads. The fragmented DNA was end-repaired, phosphorylated and “A-tailed” using the kit. Afterward, DNA fragments were ligated to adaptors of known sequences using the kit, purified using magnetic beads to remove excessive adaptors and amplified by polymerase chain amplification (PCR). The amplified libraries were purified by magnetic beads to remove excessive primers and checked for quantity and quality. The qualified libraries were denatured, cyclized, amplified by rolling circle amplification (RCA) and digested to remove double-stranded circular DNA and to obtain DNA nanoballs. The prepared DNBs were loaded onto the Patterned Array and sequenced using combinatorial probe-anchor synthesis (cPAS), which involved the polymerization of sequencing primer-anchored molecules and fluorescent probes on the DNA nanoballs, followed by the acquisition, reading and recognition of optical signals using a high-resolution imaging system to obtain individual base sequence information. Then the next cycle was performed to obtain the following base sequence information. After multiple cycles, the original sequencing data were obtained. The sequencing platform was the MGI sequencer, model DNBSEQ-T7.

### Read alignment and variant calling

Sequencing data for each material were compared and aligned to the tetraploid potato reference genome Q9 (Wang et al., 2022) using software BWA (Li and Durbin, 2009) to generate SAM files. The SAM files were reordered using SAMtools (Li et al., 2009; Li, 2011), converted to BAM files, and used to generate index files. SNPs were called using Genome Analysis Toolkit (GATK) (McKenna et al., 2010), and the variant call format (VCF) files were produced. In addition, site frequency spectrum (SFS) was applied with the call set at the population level based on MAF > 0.05 and missing rate < 0.1 to ensure that the SNPs called from the whole-genome re-sequencing data were reasonable.

### Phylogenetic and population structure analyses

SNPs with allele frequency > 0.05 and indels with deletion frequency ≤ 50% were considered high-quality and used for phylogenetic tree and population structure analyses. VCF files were converted to the HapMap format using a custom Perl script and to PLINK format files using PLINK v1.90 (http://pngu.mgh.harvard.edu/purcell/plink/). A neighbor-joining tree was constructed with TreeBeST (Li, 2006) under the p-distances model with bootstrapping (100). Principal component analysis (PCA) was performed using SNPRelate v1.18.1 (Zheng et al., 2012). First, the genetic relationship matrix was generated, and the first three eigenvectors were extracted. Second, population structure was constructed using fastStructure v1.0 (Jombart, 2008) from large SNP genotype data sets by setting K = 1 to K = 12. The appropriate K value for different subgroups was determined according to the obtained cross-validation error value. The genetic composition coefficient (Q) of each material in each subgroup was used to construct the population-genetic structure matrix. The genetic diversity (π) and population pairwise F-statistics (FST) were calculated using VCFtools (Danecek et al., 2011) software. According to Wright, FST = 0, 0 < FST < 0.05, 0.05 ≤ FST < 0.15, 0.15 ≤ FST < 0.25, 0.25 ≤ FST < 1 and FST = 1 indicate that two subgroups have no, weak, medium, strong, very strong, and complete genetic differentiation, respectively.

### Genome-wide association analysis and candidate gene identification

GWAS analysis of high-quality SNPs and indels (MAF>0.05 and HWE>0.001) was performed using the compressed mixed linear model (MLM) to identify associated loci, SNP types, and SNP locations against the reference genome (Wang et al., 2022). The total genes of each candidate region were analyzed and annotated by homology comparison with Arabidopsis to narrow down the candidate genes.

## Results

### Phenotypic identification, sequencing and variants

Among the 370 studied tetraploid potatoes, 152 were from the International Potato Center in Peru, 126 from 13 provinces or municipalities in China, 25 from Israel, Russia, Canada, Australia, USA, Netherlands, New Zealand and 67 from unknown sources (**Supplemental Table 1**). 67 potato germplasm resources lost their original source information in the process of preservation. Through the identification of phenotypic shape and genotype, it was found that these varieties had great differences (**Supplemental Tables 2**, **3**; **Supplemental Figure 1**), which could be used for GWAS. Our observation revealed that potato tuber shape and eye depth are morphological traits with continuous variations (**Supplemental Figures 2A, B**; **Supplemental Table 4**), and the median index of tube shape and eye depth is 4 (**Supplemental Figures 2C, D**). The frequency distribution histogram revealed that the observed values of potato tuber shape and potato tuber eye depth showed a normal distribution (**Supplemental Figures 2E, F**).

**Figure 1:**
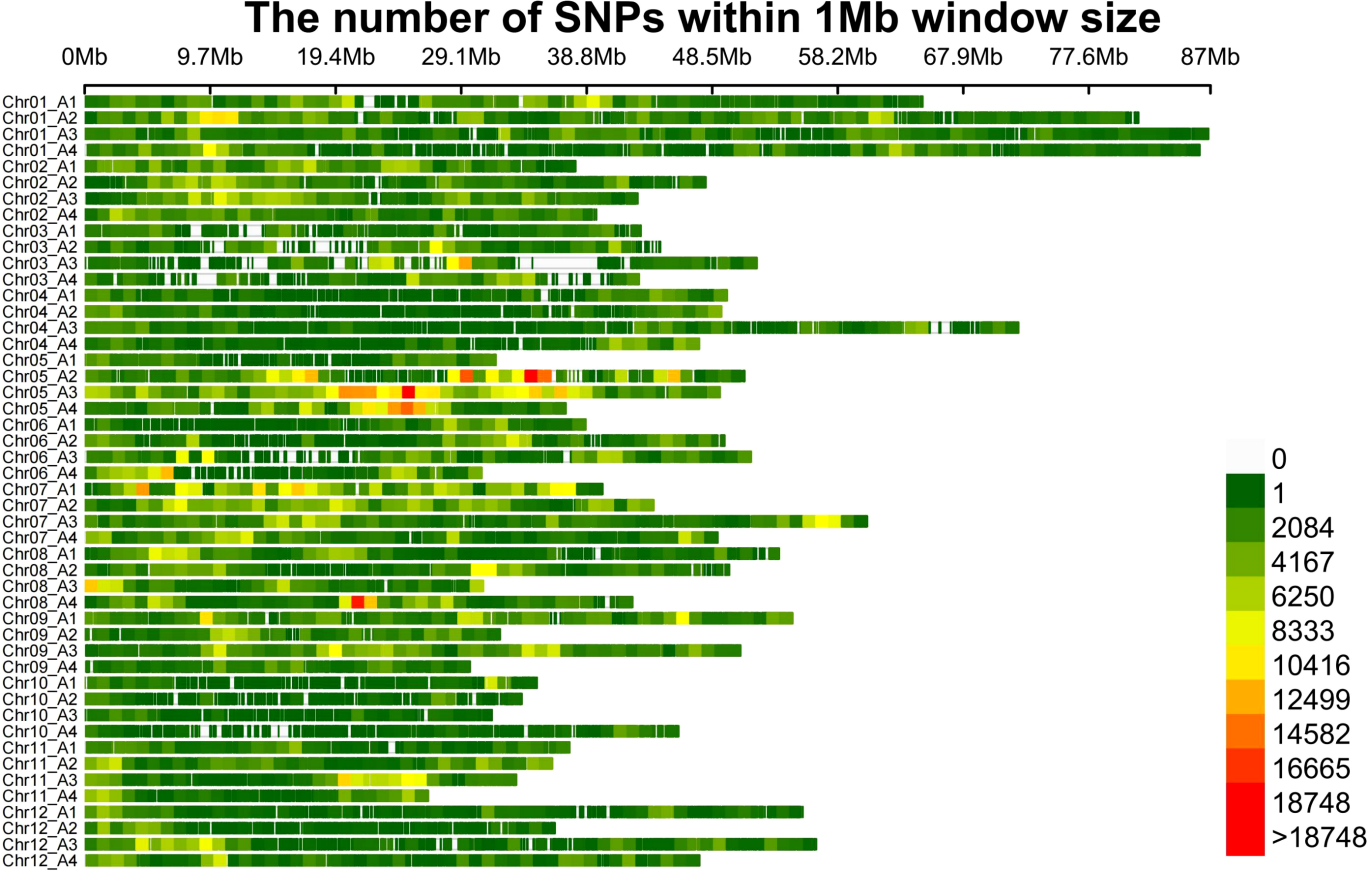
SNP distributions on 48 chromosomes of potato. The horizontal axis displays the chromosome length, and the legend insert indicates SNP density. A1-A4 represents haplotype 1 - haplotype 4 respectively.

Re-sequencing the 370 studied tetraploid potatoes generated 9.88 Tb raw data, with an average sequencing depth of ~10X. We ended up with 232,581,777 variation sites. After filtering, 4,986,690 high quality variation sites (4,535,735 SNPs, 450,955 Indels) were obtained (**Figure 1**). There were 720,431 SNPs, of which 181,309 (4.00%) were EXON and 539,122 (11.89%) were INTRON. INTERGENIC region has the most SNPs with 3,7661,148 (82.92%), followed by UTR_3_PRIME and UTR_5_PRIME with 31,949 (0.70%) and 17 388 (0.38%), respectively. There were 86,217 Indels, including 7,713 Exons (1.71%) and 78,504 introns (17.41%). INTERGENIC has the most SNPs with 354,435 (78.60%), followed by UTR_3_PRIME and UTR_5_PRIME with 6 183 (1.37%) and 3 057 (0.68%), respectively. (**Supplemental Table 5**).

### Potato population structure and genetic diversity analysis

**Figure 2:**
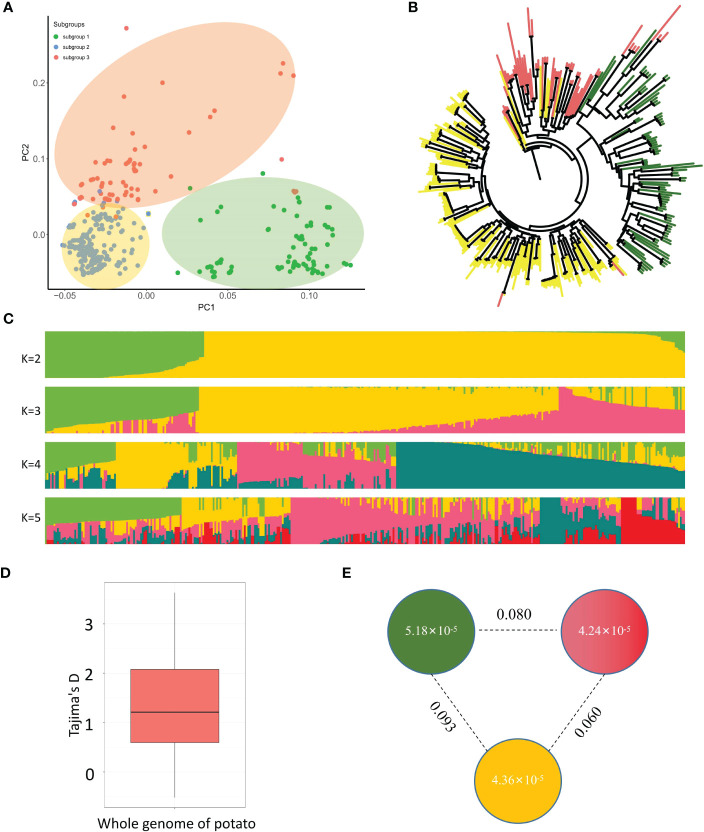
Population structure and genetic diversity analysis of the 370 studied tetraploid potatoes. **(A)** Principal component analysis of the 370 potato germplasm using high-quality SNPs and indels, where green, yellow and pink backgrounds represent Subgroup 1, Subgroup 2 and Subgroup 3, respectively. **(B)** Phylogenetic tree constructed for the 370 potato materials, where green, yellow and pink lines represent Subgroup 1, Subgroup 2 and Subgroup 3, respectively. **(C)** Population structure analysis with different cluster numbers (K=2, 3, 4 and 5) agrees with phylogenetic tree analysis. **(D)** Tajima’s D of the 370 studied tetraploid potatoes, indicating they lack rare alleles. **(E)** Nucleotide diversity (PI) and genetic differentiation coefficient (Fst) between any two of the three subgroups.

High-quality SNPs and indels were analyzed using ADMIXTURE software. Based on the possible clustering range (K) of 1-12, the cross-validation error rate (CV error) was calculated for each K value. At K=3, the CV error value is the smallest (**Supplemental Figure 3**), inferring that the most significant change occurs at K=3. Our principal component analysis revealed that the 370 studied tetraploid potato materials could be classified into three subgroups, and each could further form a cluster (**Figure 2A**). The phylogenetic tree also showed that the 370 samples are well clustered as three classes (**Figure 2B**). The tetraploid potato materials were also classified into three subgroups based on their largest Q values. **Figure 2D** lists the population structures at K=2, 3, 4 and 5. The population structure at K=3 is the best, with 89, 208 and 73 materials in Subgroup 1, Subgroup 2 and Subgroup 3, respectively (**Figure 2C**).

Moreover, the tetraploid potato population has a Tajima’s D greater than 0, indicating that rare alleles of the tetraploid potato population are present at a low frequency (lack of rare alleles) (**Figure 2D**). The genetic diversity (π) of the three subgroups was calculated using VCFtools as 5.18 × 10^-5^, 4.36 × 10^-5^ and 4.24 × 10^-5^, respectively, indicating that Subgroup 1 has the lowest genetic diversity and Subgroup 3 has the highest genetic diversity (**Figure 2E**). But the overall difference among these three subgroups is not significant. The genetic differentiation coefficient, Fst, ranges from 0 to 1 and is used to indicate the correlation between two materials. The closer the Fst is to 0, the closer the relationship between the two materials. The closer the Fst is to 1, the more distant the relationship between the two materials. In this study, the differentiation coefficient is 0.093 between Subgroup 1 and Subgroup 2, 0.080 between Subgroup 1 and Subgroup 3, and 0.060 between Subgroup 2 and Subgroup 3, indicating that the three subgroups are predominantly differentiated to a low degree (**Figure 2E**). We further performed genotype cluster analysis of the 370 tetraploid potatoes and found significant differences in genotypes among these three subgroups (**Supplemental Figure 4**).

### Genome-wide association analysis of potato tuber shape and tuber eye depth

**Figure 3:**
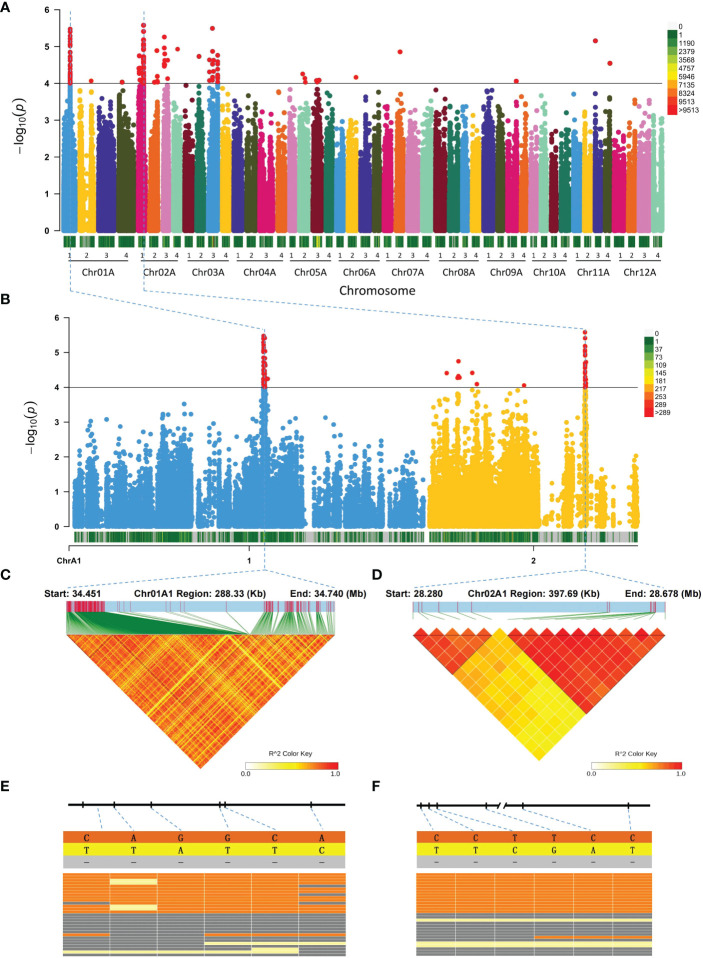
Manhattan plot and candidate regions associated with potato tuber shape. **(A)** Manhattan plot of potato tuber shapes. **(B)** Association regions on Chr01A1 and Chr02A1. **(C, D)** LD heatmaps of regions significantly associated with tuber shapes on Chr01A1 and Chr02A1, respectively. **(E)** Distribution of significant SNP markers on Chr01A1 of the round and elongated potatoes. From left to right are SNPs at Chr01A1:34451395, Chr01A1:34458899, Chr01A1:34466909, Chr01A1:34484034, Chr01A1:34484958 and Chr01A1:34503329 loci, where the upper half represents the round potatoes and lower half represents the very long potatoes, respectively. **(F)** The distribution and performance of significant SNP markers on Chr02A1 of the round and very long potatoes. From left to right are SNPs at Chr02A1:28359920, Chr02A1:28360022, Chr02A1:28360028, Chr02A1:28362877, Chr02A1:28381647 and Chr02A1:28381654, respectively.

We performed GWAS analysis of potato tuber shape using a mixed linear model with a threshold of P = 10^-4^ and plotted Manhattan (**Figure 3A**). The results revealed a total of 146 significant SNP loci **Supplemental Table 6**), of which SNP loci at the 34.451−34.740 Mb region of Chr01A1 and the 28.280−28.678 Mb region of Chr02A1 are very strongly associated (**Figure 3B**). A LD heatmap was plotted to examine the LD within the significantly associated regions and revealed highly associated SNPs within Chr01A1 and Chr02A1 regions (**Figures 3C, D**). To further explore the reliability of the SNPs related to potato tuber shape, we randomly selected 15 round potatoes and 15 very long potatoes and analyzed the distribution of six differentiated SNPs on Chr01A1 and Chr02A1 between these two types of potatoes. The results revealed that these SNPs are present on Chr01A1 (**Supplemental Table 7**) and Chr02A1 (**Supplemental Table 8**) of the potato with round tubers. The bases at the six loci are consistent among the potatoes with round tubers but different from those of the reference genome. By contrast, deletions or no mutation were found in the 15 very long potato tubers (**Figures 3E, F**), agreeing that the reference Q9 potato tuber is long. These findings further demonstrate the accuracy of our markers regarding potato tuber shape.

We calculated the LD decay of these regions and found that their LD is about 60 Kb, much larger than 1 Kb in previous reports (**Supplemental Figure 5**). By setting an interval of 60 Kb above and below the variant loci and considering a candidate gene being 10% of the gene length falling within the interval, we obtained 50 candidate genes (**Supplemental Table 9**). KEGG enrichment analysis indicated that these candidate genes are mainly enriched in starch and sucrose metabolism, arginine and proline metabolism, glycosphingolipid biosynthesis, amino sugar and nucleotide sugar metabolism and glycosaminoglycan degradation (**Supplemental Figure 6**). In addition, we found 6 transcription factors, including CO-like, ERF, FAR1, MADS and MYB (**Supplemental Table 10**), and identified 3 genes: PLATZ transcription factor, UTP-glucose-1-phosphate uridylyltransferase and FAR1 DNA-binding domain (**Supplemental Table 9**).

**Figure 4:**
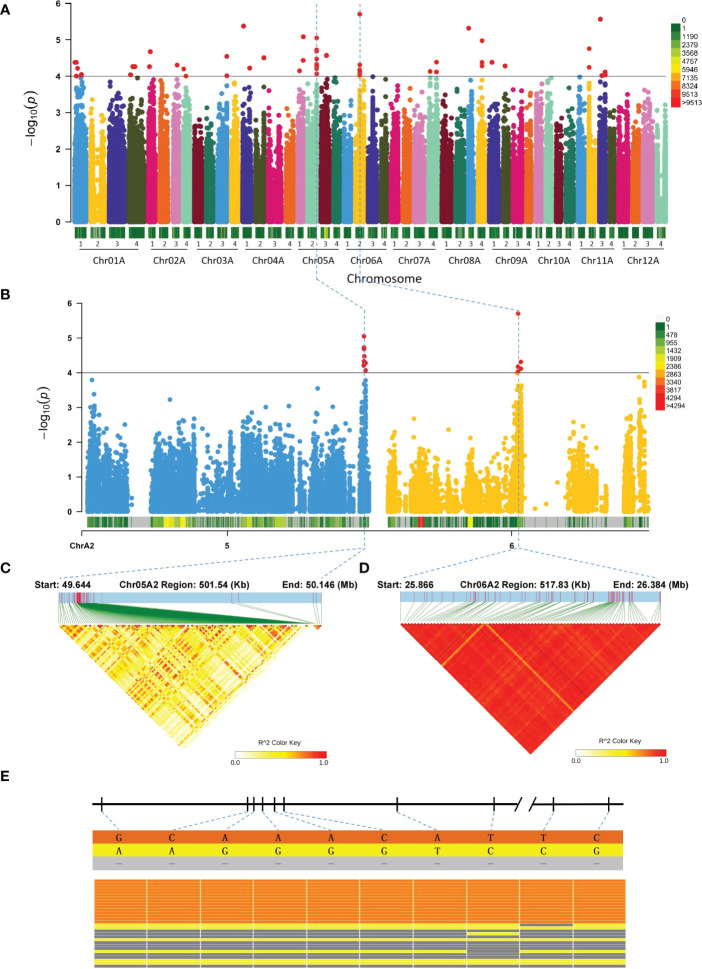
Manhattan and candidate regions associated with potato tuber eye depth. **(A)** Manhattan plot of potato tuber eye depth. **(B)** Analysis of regions associated with tuber eye depth on Chr05A2 and Chr06A2. **(C)** and **(D)** LD heatmap of regions significantly associated with tuber eye depth on Chr05A2 and Chr05A2, respectively. **(E)** Distribution and performance of significant SNP markers on Chr05A2 of potatoes with shallow and deep tuber eyes. From left to right are SNPs at Chr05A2:50031597, Chr05A2:50087233, Chr05A2:50087238, Chr05A2:50087243, Chr05A2:50087245, Chr05A2:50087357, Chr05A2:50087467 Chr05A2:50143917, Chr05A2:50385093 and Chr05A2:50385385 loci, where the upper half represents potatoes with shallow tuber eyes and the lower half represents potatoes with deep tuber eyes.

We performed GWAS analysis of potato tuber eye depth using a mixed linear model with P = 10^-4^ as the threshold and plotted Manhattan (**Figure 4A**). The results revealed a total of 53 significant SNP loci (**Supplemental Table 11**), of which SNPs in the Chr05A2:49.644−50.146 Mb and Chr06A2:25.866−26.384 Mb regions are very strongly associated (**Figure 4B**). To view the LD within the significantly associated regions, we plotted the LD heatmap and found that SNPs in the Chr05A2:49.644−50.146 Mb region are highly associated (**Figures 4C, D**). To further examine the reliability of these SNP markers for potato tuber eye depth, we randomly selected 15 potatoes with shallow tuber eyes and 15 potatoes with deep tuber eyes for analysis of 10 loci on Chr05A2 and found that all potatoes with shallow tuber eyes have deletions and alleles (**Figure 4E**; **Supplemental Table 12**).

We identified 74 candidate genes (**Supplemental Table 13**) associated with tuber eye depth and found they are mainly involved in starch and sucrose metabolism, valine, leucine and isoleucine biosynthesis, N-glycan biosynthesis, 2-oxocarboxylic acid metabolism and phenylpropanoid biosynthesis (**Supplemental Figure 7**). A total of 11 transcription factors, including B3, ERF, GATA, MYB and MADS, were also identified (**Supplemental Table 14**). We found hydrolase and methyltransferase proteins in the significantly associated region of Chr05A2 and three CYPs in the significantly associated region of Chr06A2 (**Supplemental Table 11**).

## Discussion

Potato cultivation and breeding is a hot spot and complex research area hindered by its high genetic heterozygosity and complexity. With the development of sequencing technology, the genome assembly of tetraploid potatoes has made a breakthrough. We re-sequenced 370 tetraploid potatoes, carried out GWAS analysis using a tetraploid potato cultivar Q9 from a major potato producing area in China as the reference genome, and analyzed their population structure, genetic diversity, and associations of SNPs with potato tuber shape and eye depth.

Our population structure analysis showed that the cross-validation error rate is minimal at K=3, indicating that this population can be divided into three subgroups. The genetic diversity index (π) of these 370 tetraploid potatoes is in the range from 4.24×10^-5^ to 5.18×10^-5^, much lower than that of 0.0007 for cultivated cotton (Wang et al., 2017), 0.001 for cultivated soybean (Zhou et al., 2015) 0.001 for grains (Jia et al., 2013), 0.0017 for cultivated tomato (Lin et al., 2014), and 0.0024 for cultivated rice (Huang et al., 2011), indicating that the overall diversity of the population is relatively low. Potato has undergone a long-term natural selection and artificial domestication. Thus, its population structure is very complex in different ecological environments. Population structure is an important factor causing false positives in association analysis (Mather et al., 2007). In our study, the population differentiation index of the three subgroups ranges from 0.060 to 0.093, indicating that these populations are predominantly differentiated at lower levels. LD is an important parameter for determining marker density and accuracy (Simko, 2014) in allogeneic association analysis and evolutionary population selection. It is affected by genetic and non-genetic factors. Among them, mutation and recombination are the most important, along with biological reproduction patterns, genetic drift and selection effects. In this study, the linkage distance of 60 Kb for the tetraploid potatoes is significantly greater than that of 1 Kb in previous studies (Wang et al., 2021). The possible reason for this discrepancy is that we compared all sequencing data to the 48 chromosomes of the reference tetraploid potato, which led to a significant increase in LD.

GWAS analysis is affected by many factors, including population size, structure and diversity. We selected 370 tetraploid potatoes worldwide, including 150 from the International Potato Center in Peru, to increase our study’s representativeness. Previous studies have shown that SNPs on chromosomes 1, 2, 4 and 10 are associated with round and elongated tuber shapes in potato (Lindqvist-Kreuze et al., 2015; Hara-Skrzypiec et al., 2018; Fan et al., 2022). However, there was no reference genome of tetraploid potato as described in the study before 2022, and the newly published tetraploid reference genome Q9 was used in our study. The significant tuber shapes sites found in our study were mainly in the A1 haplotypes of chromosomes 1 and 2. This difference may be related to the reference genome we used. In this study, we used the tetraploid reference genome (with 48 chromosomes), previously mainly using DM reference genome (with 12 chromosomes), and we used 370 natural populations, including almost all possible potato type traits. These findings indicate that these two regions may control potato tuber shape and are expected to be important markers for identifying potato tuber shape. We further compared the expression of twelve significant SNP loci at these two regions in the round and very long potatoes and found that the reference genome allelic bases are mainly present in the round potatoes. In contrast, deletions or bases consistent with the reference genome are present in the very long potato varieties. We screened candidate genes in the regions 60 Kb upstream and downstream of the significant SNP loci in LD pairs and found that these candidate genes are significantly enriched in the starch and sucrose metabolism pathways. The different tuber shapes are caused by differences in cell division and enlargement rates in all directions during tuber formation. We hypothesize that starch and its metabolites contribute to potato tuber formation. We found that several transcription factors in the PLATZ family are located in the hot spot of the Chr02A1:28.280−28.678 Mb region. Interestingly, studies on rice have suggested that *GL6* encodes a plant-specific PLATZ transcription factor that positively regulates seed length by promoting cell proliferation in young spikelets and seeds. Further studies have also revealed that GL6 interacts with RPC53 and *OsTFC1* and is involved in the RNA polymerase III transcriptional machinery that regulates the expression of genes involved in rice seed development (Zhou and Xue, 2020). SG6 positively regulates granule length by promoting DNA replication and cell division in glumes *via* activating the expression of cell cycle-related genes (*CDC6*, *RFC3*, *CDT1A*, *POLE2B* and *CDKB2;1*) (Wang et al., 2019). Moreover, UTP-glucose-1-phosphate uridylyltransferase, an enzyme essential to sugar metabolism, is also located in the Chr02A1:28.280−28.678 Mb region. Under certain conditions, it catalyzes the transfer of uridine from uridine triphosphate to glucose monophosphate to produce uridine diphosphate glucose and pyrophosphate. Uridine diphosphate glucose is a key metabolite in organisms’ metabolic pathways and plays a crucial role in synthesizing substances, including sucrose, cellulose and callose. Although uridine diphosphate glucose is mainly generated through catalysis of UTP-glucose-1-phosphate-uridyltransferase, the reaction is related to the metabolism of downstream disaccharides and polysaccharides. Our study found that Far-red impaired response 1 (*FAR1*) is located at Chr02A1:28387314−28398490. The protein contains a DNA-binding domain and belongs to a transposase-derived class of transcription factors capable of direct activation the expression of far-red light gene *FHY1/FHL*, an important regulatory player in plant starch anabolism and energy deprivation triggered by carbon starvation (Casal, 2000). Similar to the previous findings, significant SNP loci were present in all chromosomes 1, 3, 4, 5 and 11 in our study (Hara-Skrzypiec et al., 2018). Our GWAS analysis for potato tuber eye depth revealed a robust association between Chr05A2:49.644-50.146 Mb and Chr06A2:25.866−26.384 Mb regions, both of which are expected to be important markers for identifying tuber eye depth in tetraploid potatoes. The hydrolase and methyltransferase genes are present in the Chr05A2:49.644−50.146 Mb region. Their genotypes will greatly affect tuber eye depth, which is also influenced by environmental factors (Hara-Skrzypiec et al., 2018). The Chr06A2:25.866-26.384 Mb region contains three CYP genes. CYPs regulate plant growth and development mainly *via* the growth hormone transport and signaling pathways (Jackson and Söll, 1999; Yoon et al., 2015) by affecting the translocation of growth hormone transporters and expression of growth hormone regulatory genes. We believe that CYPs play an important role in regulating potato tuber eye depth.

The study analyzed the population structure and genetic diversity of 370 tetraploid potatoes, determined their LD and identified the possible chromosomal regions and candidate genes that affect potato tuber shape and eye depth. The next step is to verify the candidate genes in extreme samples and genetic populations. These findings enriched GWAS analyses and provided insights for future candidate gene mining in tetraploid potatoes.

## Data availability statement

The data presented in the study are deposited in the National Genomics Data Center (http://bigd.big.ac.cn/) repository, accession number PRJCA011806.

## Author contributions

LZ, ZX and FW conceived and managed the experiments. LZ, KD, XD, MH and TN conducted the experiments work. LZ, FW, CX, SJ, CZ and ZX analyzed the experimental results. LZ and MZ wrote the manuscript. ZX, FW and JW reviewed and contributed to improve it and revised the last version of the manuscript. All authors contributed to the article and approved the submitted version.
