# Supplementary material for: Insights into the genetic determination of tuber shape and eye depth in potato natural population based on autotetraploid potato genome
Source: Front Plant Sci. 2023 Mar 28;14:1080666. doi: 10.3389/fpls.2023.1080666 (PMC10086151; doi:10.3389/fpls.2023.1080666)
Supplement: Supplementary file 1 [file DataSheet_1.docx]

**Supplemental data**

**
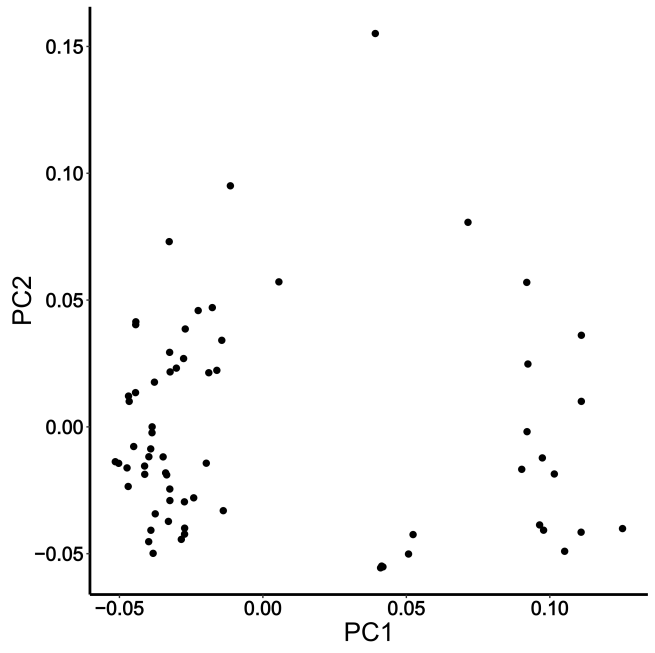
**

**Supplemental Figure 1** Principal component analysis of the 67 potato varieties from unknown sources using high-quality SNPs and indels.


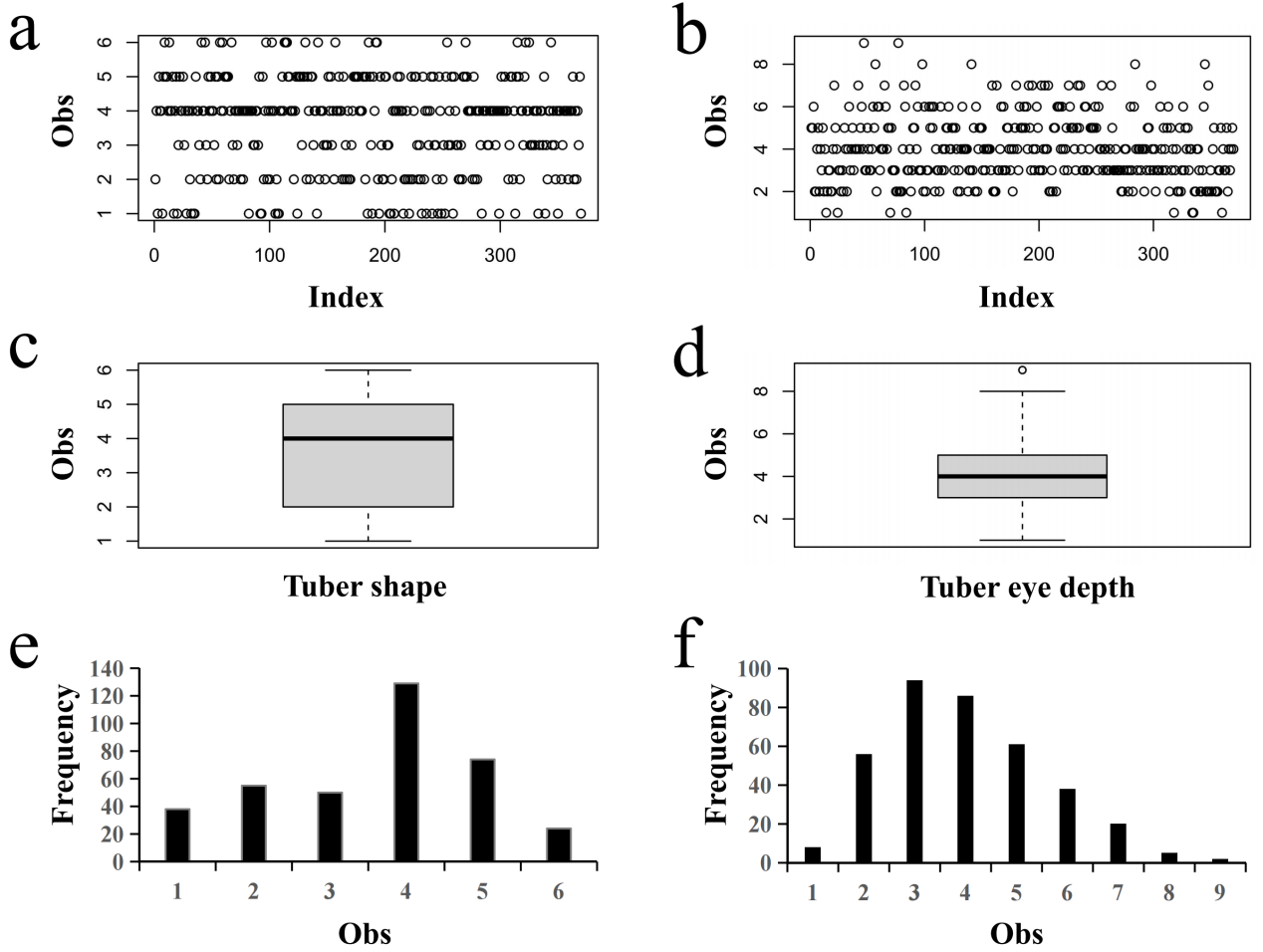


**Supplemental Figure 2** Distribution of potato tuber shape and eye depth among the 370 tetraploid potatoes

**Supplemental Figure 3** Cross-validation error rate at each K value


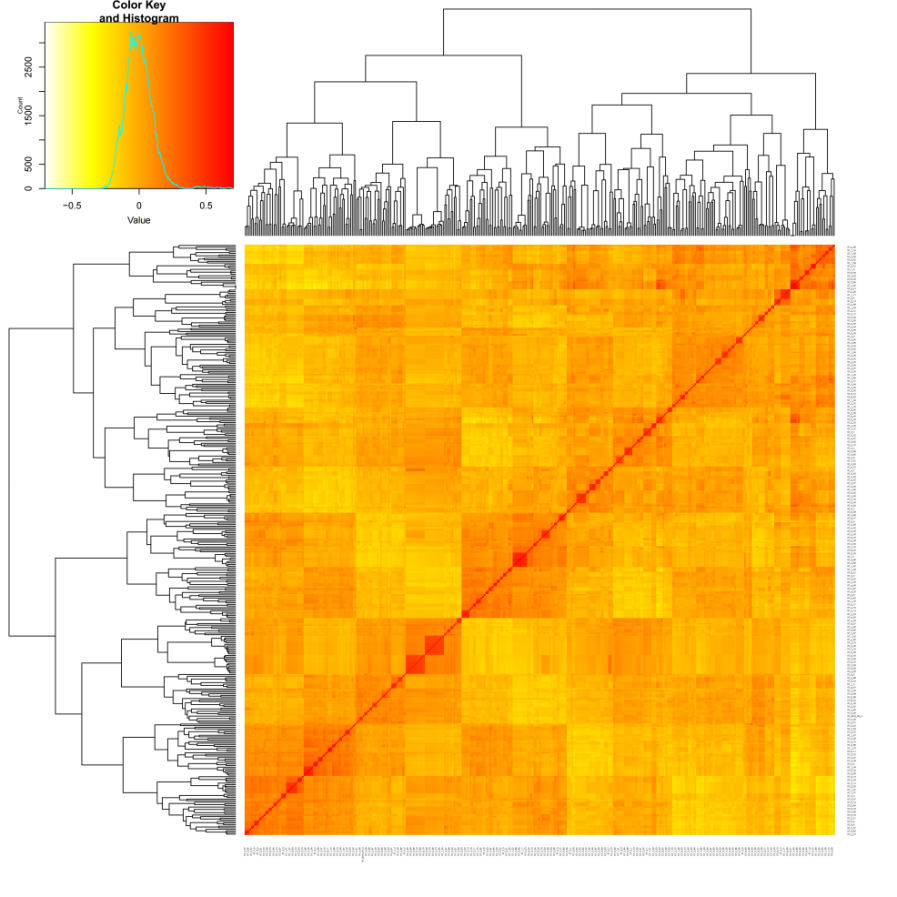


**Supplemental Figure 4** Genotype cluster heatmap of 370 tetraploid potatoes


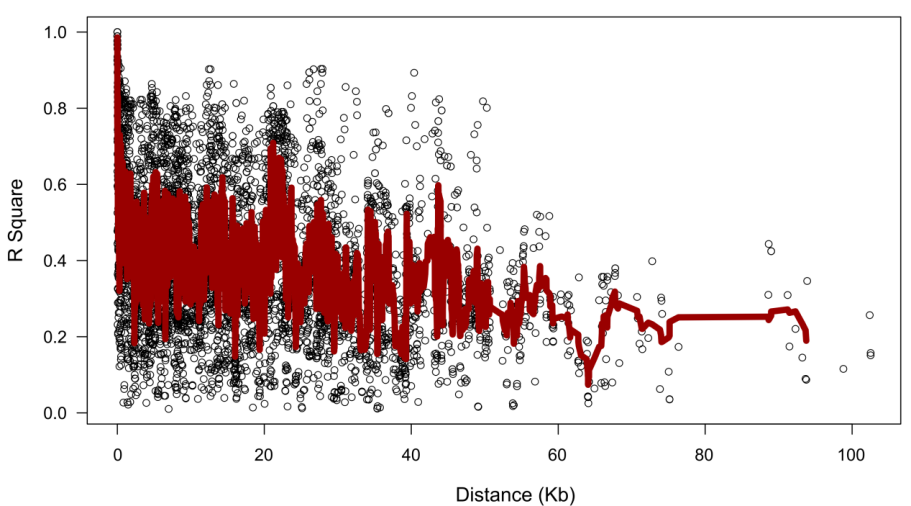


**Supplemental Figure 5** LD of 370 tetraploid potatoes


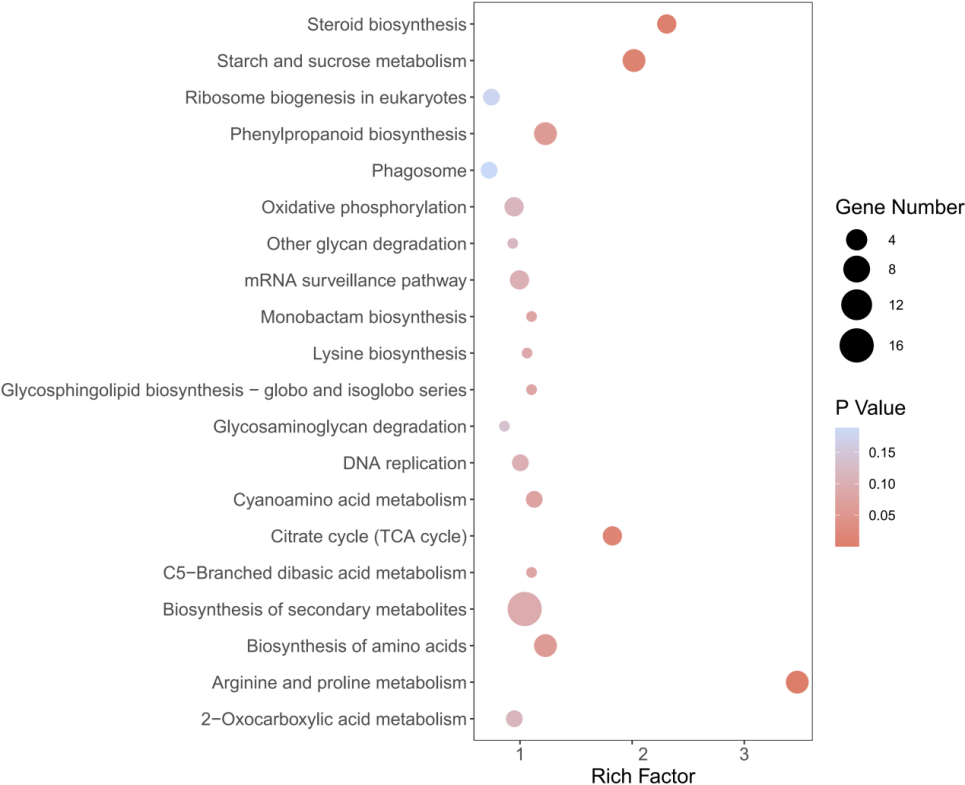


**Supplemental Figure 6** KEGG enrichment analysis of the candidate genes controlling potato tuber shape


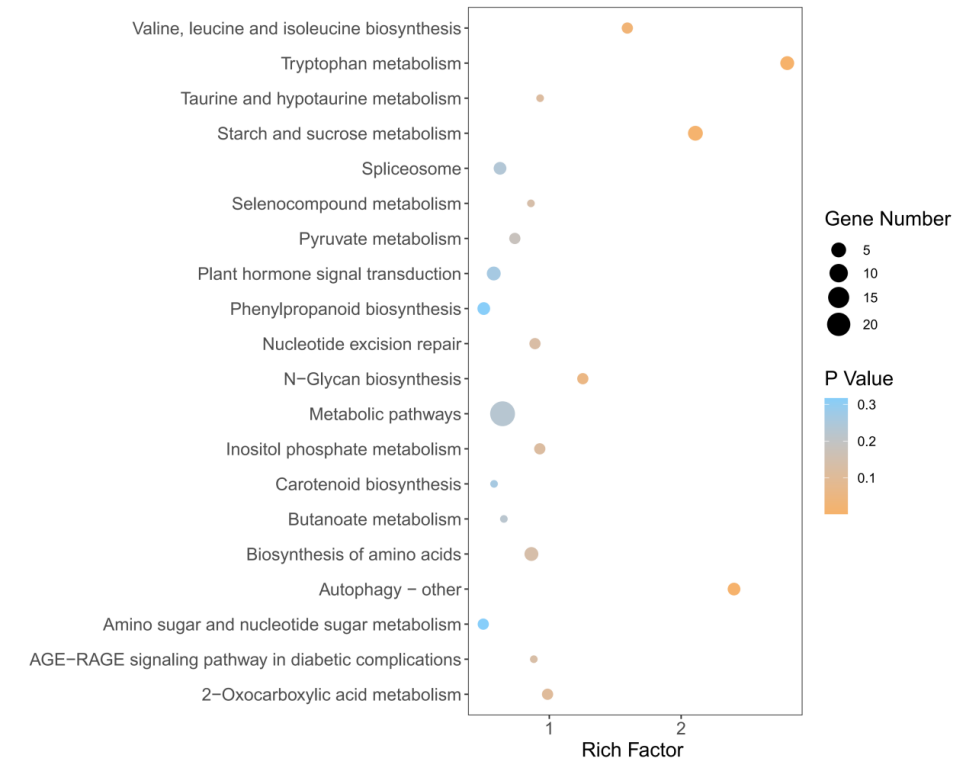


**Supplemental Figure 7** KEGG enrichment analysis of the candidate genes controlling potato tuber eye depth
