# Supplementary material for: Insights into the genetic determination of tuber shape and eye depth in potato natural population based on autotetraploid potato genome
Source: Front Plant Sci. 2023 Mar 28;14:1080666. doi: 10.3389/fpls.2023.1080666 (PMC10086151; doi:10.3389/fpls.2023.1080666)
Supplement: Supplementary file 2 [file Table_1.docx]

**Supplemental data**

**Supplemental Table 1** Source of 370 tetraploid potatoes

| ID | Name | Sourse | ID | Name | Sourse |
| --- | --- | --- | --- | --- | --- |
| ST_1_10 | 124 | unknown | ST_4_12 | 5p1-1 | China |
| ST_1_12 | 11 | unknown | ST_4_13 | Russ2 | Russia |
| ST_1_13 | Katahdin×ke | China | ST_4_14 | DR-4 | China |
| ST_1_14 | Ke5-11 | China | ST_4_16 | Feixing.2 | unknown |
| ST_1_15 | e5-1 | Peru | ST_4_17 | Shiyin08—1 | unknown |
| ST_1_16 | R16 | Peru | ST_4_18 | E8 | Peru |
| ST_1_17 | Hua525.1 | China | ST_4_2 | Perou08-2 | Peru |
| ST_1_18 | Jia7 | Canada | ST_4_20 | 393882.44 | Peru |
| ST_1_21 | Zhong9 | China | ST_4_21 | Cy-Ⅱ-9 | Peru |
| ST_1_22 | D1 | unknown | ST_4_22 | UP-3 | China |
| ST_1_23 | Ke9912-3 | China | ST_4_23 | DR-3 | China |
| ST_1_26 | Ke200373-13 | China | ST_4_25 | manana | unknown |
| ST_1_27 | Qing5.1 | China | ST_4_26 | 139 | unknown |
| ST_1_28 | Ke8 | China | ST_4_27 | Norship | unknown |
| ST_1_29 | 720018.1 | unknown | ST_4_28 | Jiadabai | Canada |
| ST_1_3 | Zhong227 | China | ST_4_29 | E19.1 | Peru |
| ST_1_31 | B18.1 | Peru | ST_4_3 | E65 | Peru |
| ST_1_34 | B11 | Peru | ST_4_30 | Fuke76 | China |
| ST_1_35 | Isread2.1 | Israel | ST_4_31 | IP84008.4.1 | Peru |
| ST_1_36 | CIP09-6 | Peru | ST_4_33 | Youxiao | unknown |
| ST_1_37 | BH2-5 | China | ST_4_36 | Israel | Israel |
| ST_1_38 | E12.1 | Peru | ST_4_37 | IP84008.4.2 | Peru |
| ST_1_39 | F20 | Peru | ST_4_38 | Long8 | China |
| ST_1_4 | CIP09-2 | Peru | ST_4_39 | ACD338 | unknown |
| ST_1_40 | Isread1 | Israel | ST_4_4 | 183 | unknown |
| ST_1_41 | 5p2-1 | China | ST_4_40 | Isread2.2 | Israel |
| ST_1_42 | B5 | Peru | ST_4_41 | UK3 | unknown |
| ST_1_44 | DR-9.1 | China | ST_4_43 | Cy-Ⅲ-18 | Peru |
| ST_1_45 | BE13-7 | Russia | ST_4_44 | DY4-5 | China |
| ST_1_46 | B1 | Peru | ST_4_45 | E80 | Peru |
| ST_1_47 | B3 | Peru | ST_4_46 | Kajin | China |
| ST_1_48 | RH1 | unknown | ST_4_47 | E76 | Peru |
| ST_1_5 | Ke6108-17 | China | ST_4_48 | 2340 | unknown |
| ST_1_50 | BE13-4 | Russia | ST_4_49 | IP84008.3 | Peru |
| ST_1_53 | DR-6.1 | China | ST_4_5 | 348 | unknown |
| ST_1_55 | 5P2-6.1 | China | ST_4_51 | Jemsay | China |
| ST_1_57 | T1 | Peru | ST_4_52 | NZY | New Zealand |
| ST_1_58 | Zhongshu227 | China | ST_4_53 | 758792 | Peru |
| ST_1_59 | 283 | unknown | ST_4_54 | F8701.2 | China |
| ST_1_61 | ZhongC901 | China | ST_4_56 | 268 | unknown |
| ST_1_62 | B14.1 | Peru | ST_4_57 | E14.1 | Peru |
| ST_1_63 | Qing10 | China | ST_4_58 | C88 | China |
| ST_1_64 | Huayun | unknown | ST_4_6 | E22 | Peru |
| ST_1_65 | CIP09-16 | Peru | ST_4_61 | Hermes | unknown |
| ST_1_67 | 5p2-3 | China | ST_4_62 | LC-98 | unknown |
| ST_1_68 | T2 | Peru | ST_4_63 | Eromnede | unknown |
| ST_1_69 | CIP09-10 | Peru | ST_4_64 | BDRA | unknown |
| ST_1_7 | Ningshu1.1 | China | ST_4_65 | Zhukefu×FL1867 | China |
| ST_1_70 | NE303.1 | China | ST_4_67 | 63 | unknown |
| ST_1_71 | R1R3.1 | Peru | ST_4_7 | AKCFC | unknown |
| ST_1_73 | W | Peru | ST_4_72 | Fedori | unknown |
| ST_1_75 | B19 | Peru | ST_4_73 | 4(2012) | unknown |
| ST_1_76 | E62 | Peru | ST_4_8 | R9.1 | Peru |
| ST_1_77 | D8 | Peru | ST_4_83 | Pepo416.2 | Peru |
| ST_1_79 | CIP09-27 | Peru | ST_4_85 | R10 | Peru |
| ST_1_8 | Jklway | Peru | ST_4_86 | Xingjia1 | China |
| ST_1_9 | CIP09-18 | Peru | ST_4_88 | D13 | Peru |
| ST_2_1 | R3R4 | Peru | ST_4_89 | JD202461-199 | China |
| ST_2_10 | Yunshu501 | China | ST_4_9 | 288 | unknown |
| ST_2_14 | Rigao | unknown | ST_4_91 | P5.1 | Australia |
| ST_2_15 | D23 | Peru | ST_4_92 | R1R2 | Peru |
| ST_2_16 | Jizhangshu8 | China | ST_4_93 | Supiror | China |
| ST_2_17 | 5p2-6.2 | China | ST_4_94 | Jia3 | Canada |
| ST_2_18 | 4p2-2 | China | ST_4_95 | 5P1-5 | China |
| ST_2_19 | Ke6 | China | ST_4_97 | UK6 | unknown |
| ST_2_2 | D16 | Peru | ST_4_98 | Hongwenbai | United States |
| ST_2_20 | Ke3 | China | ST_4_99 | UK12 | unknown |
| ST_2_21 | Zhongshu19 | China | ST_5_1 | DT033 | China |
| ST_2_22 | 10 | China | ST_5_10 | E12.3 | Peru |
| ST_2_24 | R2 | Peru | ST_5_11 | W15 | Peru |
| ST_2_25 | Zhongshu18 | China | ST_5_12 | BoⅡ.2 | unknown |
| ST_2_26 | Bulaodao | China | ST_5_13 | DT02 | China |
| ST_2_27 | Maiken1 | China | ST_5_14 | A2.1 | Peru |
| ST_2_28 | Shenyanwo | China | ST_5_15 | Ninglu0.1 | China |
| ST_2_3 | Ke21 | China | ST_5_16 | Qing05-12-6 | China |
| ST_2_30 | W5 | Peru | ST_5_17 | Ningshu1.2 | China |
| ST_2_31 | Feixing.1 | unknown | ST_5_18 | BoS | China |
| ST_2_32 | Cy-Ⅱ-19 | Peru | ST_5_19 | DR-6.2 | China |
| ST_2_33 | B10 | Peru | ST_5_2 | DR-9.2 | China |
| ST_2_34 | E52 | Peru | ST_5_20 | LT-7 | China |
| ST_2_35 | B16 | Peru | ST_5_21 | BH2-4 | China |
| ST_2_39 | F21 | Peru | ST_5_22 | Hu9052—2 | China |
| ST_2_4 | BoⅡ.1 | unknown | ST_5_23 | Fuke212 | China |
| ST_2_40 | B4.1 | Peru | ST_5_24 | E60 | Peru |
| ST_2_41 | Zhengshu8 | China | ST_5_26 | Cy-Ⅲ-19 | Peru |
| ST_2_43 | 11(Belleile) | unknown | ST_5_28 | R13 | Peru |
| ST_2_44 | F12 | Peru | ST_5_29 | ACD342 | unknown |
| ST_2_45 | B6 | Peru | ST_5_3 | CIP09-5 | Peru |
| ST_2_46 | B24 | Peru | ST_5_30 | T6.2 | unknown |
| ST_2_48 | 8 | China | ST_5_31 | 258 | unknown |
| ST_2_49 | E12.2 | Peru | ST_5_32 | B18.2 | Peru |
| ST_2_5 | Ke200373 | China | ST_5_33 | E20 | Peru |
| ST_2_50 | B13 | Peru | ST_5_34 | Ning16 | China |
| ST_2_52 | F17 | Peru | ST_5_35 | E5 | Peru |
| ST_2_53 | T6.1 | Peru | ST_5_36 | Tuo175 | China |
| ST_2_54 | NE303.2 | China | ST_5_37 | Zhongshu21 | China |
| ST_2_55 | B9 | Peru | ST_5_40 | Sebage | China |
| ST_2_56 | E99 | Peru | ST_5_42 | S8 | Peru |
| ST_2_58 | Datongliwaihuang | China | ST_5_43 | E14.2 | Peru |
| ST_2_62 | Cy-Ⅱ-25 | Peru | ST_5_44 | A1 | Peru |
| ST_2_63 | Jizhangshu3 | China | ST_5_45 | E23 | Peru |
| ST_2_65 | JK5 | Peru | ST_5_47 | Chinese Red | China |
| ST_2_68 | 6 | China | ST_5_5 | Hu5（115） | China |
| ST_2_7 | P6 | Australia | ST_5_52 | KW-29 | Peru |
| ST_2_71 | F13 | Peru | ST_5_53 | S17 | Peru |
| ST_2_72 | CIP09-3 | Peru | ST_5_56 | CIP10-1.2 | Peru |
| ST_2_73 | 5p2-4 | China | ST_5_57 | Hu5 | China |
| ST_2_75 | CIP09-9 | Peru | ST_5_58 | B2 | Peru |
| ST_2_76 | F18 | Peru | ST_5_59 | Qingzangxuan2 | China |
| ST_2_78 | CIP10-1.1 | Peru | ST_5_6 | Minshu2 | China |
| ST_2_79 | BE13-3 | Russia | ST_5_60 | Yanshu3 | China |
| ST_2_8 | Bf1-1 | Peru | ST_5_62 | Tian0527-4 | China |
| ST_2_80 | Qingshu9 | China | ST_5_63 | A2.2 | Peru |
| ST_2_9 | 71 | unknown | ST_5_65 | Dd-Ⅰ-16 | Peru |
| ST_3_1 | Cy-Ⅲ-2 | Peru | ST_5_67 | S1 | Peru |
| ST_3_10 | 2（2012） | unknown | ST_5_68 | S25.2 | Peru |
| ST_3_11 | W38 | Peru | ST_5_69 | N01 | unknown |
| ST_3_12 | 182 | unknown | ST_5_7 | W3 | Peru |
| ST_3_13 | Pepo416.1 | Peru | ST_5_70 | D11 | Peru |
| ST_3_14 | 4p1-2 | China | ST_5_72 | KW-11 | Peru |
| ST_3_15 | Zhong4.1 | China | ST_5_74 | S5 | Peru |
| ST_3_16 | Perou08-3 | Peru | ST_5_78 | S.st | Peru |
| ST_3_17 | Dongnong303 | China | ST_5_79 | Ninglu0.2 | China |
| ST_3_18 | Perou08-1 | Peru | ST_5_8 | Anuile | unknown |
| ST_3_19 | F8701.1 | China | ST_5_81 | KW-40 | Peru |
| ST_3_2 | Zihuabai | China | ST_5_82 | BE13-11 | Russia |
| ST_3_20 | Zhong113 | China | ST_5_84 | Y3 | Peru |
| ST_3_21 | 8089-115 | unknown | ST_5_87 | B27 | Peru |
| ST_3_22 | S25.1 | Peru | ST_5_88 | F4 | Peru |
| ST_3_23 | Bf1-4 | Peru | ST_5_89 | BH1-1 | China |
| ST_3_24 | H24 | unknown | ST_5_9 | Nunone | unknown |
| ST_3_25 | 5p1-4 | China | ST_5_91 | Isread2.3 | Israel |
| ST_3_27 | Zhong4.2 | China | ST_5_92 | 5p2-5 | China |
| ST_3_28 | 720018.2 | unknown | ST_6_1 | Zaodabai.2 | China |
| ST_3_29 | Shenzhong222 | China | ST_6_11 | Jiabai | Canada |
| ST_3_3 | E5-2 | Peru | ST_6_12 | A6 | Peru |
| ST_3_31 | Bf1-2 | Peru | ST_6_13 | E41 | Peru |
| ST_3_33 | Qing8 | China | ST_6_14 | R1R3.2 | Peru |
| ST_3_35 | Dd-Ⅱ-6 | Peru | ST_6_15 | B14.2 | Peru |
| ST_3_36 | NTB.1 | unknown | ST_6_17 | E18 | Peru |
| ST_3_37 | H2 | unknown | ST_6_18 | Eroracade | unknown |
| ST_3_38 | Y1 | Peru | ST_6_19 | 4p1-3 | China |
| ST_3_39 | ZhongA9215-84 | China | ST_6_2 | Zhongda1 | China |
| ST_3_4 | E73.1 | Peru | ST_6_20 | B4.2 | Peru |
| ST_3_40 | Hu8212-3.1 | China | ST_6_21 | E73.2 | Peru |
| ST_3_41 | Atlantic | United States | ST_6_22 | E39 | Peru |
| ST_3_43 | nonona | unknown | ST_6_23 | BE13-10 | Russia |
| ST_3_47 | 36221 | unknown | ST_6_24 | E42 | Peru |
| ST_3_5 | E93 | Peru | ST_6_25 | Taihong | China |
| ST_3_51 | IVP101 | China | ST_6_26 | LT-5 | China |
| ST_3_52 | 38909 | unknown | ST_6_27 | Felix Rita | Netherlands |
| ST_3_53 | UK9 | unknown | ST_6_28 | A11 | Peru |
| ST_3_59 | Zaodabai.1 | China | ST_6_3 | Ke1 | China |
| ST_3_6 | Zangxuan | China | ST_6_32 | A3 | Peru |
| ST_3_60 | Hu8212-3.2 | China | ST_6_33 | R9.2 | Peru |
| ST_3_61 | ACD151 | unknown | ST_6_34 | Zhangshu1 | China |
| ST_3_63 | Hua525.2 | China | ST_6_36 | E19.2 | Peru |
| ST_3_64 | Cy-Ⅱ－７ | Peru | ST_6_38 | Taihe | China |
| ST_3_65 | Ｗ２０ | Peru | ST_6_39 | B7 | Peru |
| ST_3_66 | Bf1-3 | Peru | ST_6_4 | Yan97—7 | China |
| ST_3_68 | Ningshu0.1 | China | ST_6_40 | KW-59 | Peru |
| ST_3_7 | NTB.2 | unknown | ST_6_43 | 4p2-8.1 | China |
| ST_3_70 | 426 | unknown | ST_6_45 | 06-26-7 | unknown |
| ST_3_74 | 210(2012) | unknown | ST_6_48 | KW-22 | Peru |
| ST_3_76 | Qing168 | China | ST_6_49 | KW-56 | Peru |
| ST_3_77 | DB（Df） | unknown | ST_6_50 | 4p2-8.2 | China |
| ST_3_8 | Chunshu1 | China | ST_6_51 | S115 | Peru |
| ST_3_83 | Ziyun | unknown | ST_6_52 | KW-41 | Peru |
| ST_3_87 | Ke13 | China | ST_6_53 | KW-24 | Peru |
| ST_3_88 | D568 | Peru | ST_6_54 | UK8 | unknown |
| ST_3_89 | Qing5.2 | China | ST_6_57 | Dd-Ⅱ-17 | Peru |
| ST_3_9 | Jiadahong | Canada | ST_6_58 | Jin2004-4-14 | China |
| ST_3_90 | W28 | Peru | ST_6_63 | Dd-Ⅱ-11 | Peru |
| ST_3_91 | CIP09-1 | Peru | ST_6_67 | A4 | Peru |
| ST_3_92 | CIP09-12 | Peru | ST_6_7 | Jiabai7 | Canada |
| ST_3_93 | UK7 | unknown | ST_6_8 | 222 | unknown |
| ST_3_94 | Maiyi233 | unknown | ST_6_81 | L9901-132 | unknown |
| ST_3_95 | E66 | Peru | ST_6_83 | D4 | Peru |
| ST_3_96 | Qingshu3 | China | ST_6_84 | P5.2 | Australia |
| ST_3_97 | Weishu1 | China | ST_6_85 | BH2-1.1 | China |
| ST_3_98 | 4P2-9 | China | ST_6_86 | D14 | Peru |
| ST_3_99 | D12 | Peru | ST_6_88 | BH2-1.2 | China |
| ST_4_1 | Dinila | unknown | ST_6_89 | Isread3 | Israel |
| ST_4_10 | DR-8 | China | ST_6_9 | Bai5-2 | China |
| ST_4_11 | E94 | Peru | ST_CH_3 | CH_3 | Peru |

**Supplemental Table 2** Phenotypic identification of 67 potato varieties from unknown sources

| ID | Tuber meat color | Tuber skin color | Tuber eye base color | Compound leaf size | Number of compound leaf lobules | Size of parietal lobule | Plant height |
| --- | --- | --- | --- | --- | --- | --- | --- |
| ST_1_10 | 3 | 4 | 3 | 6 | 6 | 6 | 7 |
| ST_1_12 | 5 | 3 | 2 | 3 | 7 | 3 | 5 |
| ST_1_22 | 2 | 2 | 2 | 4 | 4 | 4 | 4 |
| ST_1_29 | 2 | 1 | 1 | 6 | 4 | 5 | 3 |
| ST_1_48 | 3 | 3 | 2 | 4 | 5 | 4 | 4 |
| ST_1_59 | 7 | 2 | 2 | 5 | 4 | 5 | 3 |
| ST_1_64 | 9 | 6 | 3 | 7 | 4 | 6 | 6 |
| ST_2_14 | 4 | 2 | 2 | 6 | 5 | 6 | 3 |
| ST_2_31 | 2 | 4 | 1 | 4 | 4 | 5 | 4 |
| ST_2_4 | 1 | 1 | 2 | 6 | 3 | 6 | 3 |
| ST_2_43 | 5 | 2 | 2 | 6 | 5 | 6 | 5 |
| ST_2_9 | 3 | 2 | 2 | 4 | 2 | 5 | 2 |
| ST_3_10 | 2 | 1 | 2 | 4 | 2 | 4 | 2 |
| ST_3_12 | 4 | 2 | 2 | 5 | 4 | 5 | 5 |
| ST_3_21 | 5 | 2 | 2 | 6 | 4 | 6 | 4 |
| ST_3_24 | 4 | 1 | 2 | 6 | 8 | 3 | 7 |
| ST_3_28 | 3 | 2 | 2 | 4 | 3 | 3 | 2 |
| ST_3_36 | 3 | 2 | 2 | 6 | 5 | 5 | 7 |
| ST_3_37 | 4 | 2 | 2 | 4 | 4 | 3 | 3 |
| ST_3_43 | 2 | 2 | 1 | 6 | 3 | 5 | 2 |
| ST_3_47 | 3 | 2 | 1 | 3 | 3 | 2 | 3 |
| ST_3_52 | 3 | 2 | 1 | 3 | 2 | 2 | 3 |
| ST_3_53 | 4 | 2 | 1 | 4 | 4 | 5 | 5 |
| ST_3_61 | 5 | 2 | 2 | 3 | 2 | 3 | 5 |
| ST_3_7 | 3 | 2 | 2 | 5 | 4 | 6 | 7 |
| ST_3_70 | 4 | 4 | 3 | 2 | 2 | 3 | 2 |
| ST_3_74 | 4 | 3 | 3 | 2 | 3 | 1 | 2 |
| ST_3_77 | 2 | 2 | 3 | 5 | 6 | 3 | 4 |
| ST_3_83 | 4 | 6 | 4 | 5 | 7 | 3 | 7 |
| ST_3_93 | 3 | 2 | 3 | 5 | 2 | 3 | 3 |
| ST_3_94 | 5 | 2 | 3 | 5 | 5 | 5 | 5 |
| ST_4_1 | 4 | 2 | 2 | 4 | 3 | 5 | 5 |
| ST_4_16 | 5 | 2 | 3 | 5 | 5 | 5 | 5 |
| ST_4_17 | 3 | 2 | 2 | 5 | 5 | 5 | 5 |
| ST_4_25 | 4 | 2 | 2 | 5 | 4 | 5 | 3 |
| ST_4_26 | 3 | 2 | 2 | 8 | 5 | 7 | 6 |
| ST_4_27 | 5 | 2 | 3 | 2 | 6 | 3 | 1 |
| ST_4_33 | 3 | 2 | 1 | 4 | 3 | 4 | 2 |
| ST_4_39 | 4 | 3 | 2 | 4 | 3 | 4 | 2 |
| ST_4_4 | 4 | 2 | 2 | 6 | 5 | 6 | 3 |
| ST_4_41 | 6 | 7 | 2 | 4 | 3 | 4 | 3 |
| ST_4_48 | 4 | 3 | 2 | 5 | 4 | 4 | 4 |
| ST_4_5 | 4 | 2 | 2 | 6 | 4 | 6 | 3 |
| ST_4_56 | 4 | 2 | 2 | 6 | 6 | 6 | 4 |
| ST_4_61 | 2 | 2 | 2 | 3 | 3 | 3 | 2 |
| ST_4_62 | 1 | 2 | 2 | 5 | 2 | 4 | 2 |
| ST_4_63 | 4 | 2 | 2 | 3 | 3 | 3 | 2 |
| ST_4_64 | 5 | 2 | 2 | 6 | 6 | 6 | 4 |
| ST_4_67 | 3 | 2 | 2 | 5 | 6 | 5 | 3 |
| ST_4_7 | 4 | 2 | 2 | 6 | 7 | 7 | 6 |
| ST_4_72 | 5 | 2 | 2 | 5 | 4 | 5 | 3 |
| ST_4_73 | 5 | 2 | 2 | 5 | 2 | 5 | 2 |
| ST_4_9 | 4 | 2 | 2 | 6 | 3 | 7 | 6 |
| ST_4_97 | 2 | 2 | 2 | 4 | 3 | 4 | 3 |
| ST_4_99 | 2 | 2 | 3 | 6 | 4 | 6 | 3 |
| ST_5_12 | 3 | 2 | 2 | 4 | 4 | 4 | 2 |
| ST_5_29 | 4 | 4 | 2 | 5 | 4 | 5 | 3 |
| ST_5_30 | 5 | 2 | 2 | 6 | 4 | 6 | 4 |
| ST_5_31 | 5 | 2 | 2 | 5 | 5 | 3 | 3 |
| ST_5_69 | 2 | 2 | 1 | 5 | 4 | 5 | 5 |
| ST_5_8 | 4 | 1 | 2 | 4 | 3 | 3 | 4 |
| ST_5_9 | 2 | 1 | 2 | 6 | 6 | 6 | 2 |
| ST_6_18 | 4 | 2 | 2 | 3 | 3 | 3 | 3 |
| ST_6_45 | 2 | 1 | 1 | 4 | 5 | 5 | 6 |
| ST_6_54 | 2 | 1 | 2 | 5 | 2 | 5 | 6 |
| ST_6_8 | 1 | 1 | 2 | 6 | 5 | 7 | 7 |
| ST_6_81 | 5 | 2 | 2 | 5 | 5 | 5 | 4 |

Traits were identified according to the national standards of the People's Republic of China (GB/T 19557.28-2018; <https://openstd.samr.gov.cn/bzgk/gb/)>, also in Supplemental Table 3.

**Supplemental Table 3** Identification standards of potato phenotype

| Agronomic Trait | | The Name of Standard Cultivar | Index |
| --- | --- | --- | --- |
| Tuber meat color | White | Russet Burbank | 1 |
|  | Milky white | Desiree | 2 |
|  | Light yellow | Zhongshu 5 | 3 |
|  | Medium yellow | Ningshu 1 | 4 |
|  | Dark yellow | Lt-7 | 5 |
|  | Red | Hongmeigui 1 | 6 |
|  | Partial red | Yunshu603 | 7 |
|  | Blue | Heimeigui 1 | 8 |
|  | Partial blue | Yunshu602 | 9 |
| Tuber skin color | Light yellow | Zhongshu 5 | 1 |
|  | Yellow | Dongnong303 | 2 |
|  | Light red | Norland | 3 |
|  | Red | Hongying | 4 |
|  | Partial red | Red Warba | 5 |
|  | Blue | Brigus | 6 |
|  | Partial blue | Kestrel | 7 |
|  | Reddish-brown | Umatilla Russet | 8 |
| Tuber eye base color | White | Nadine | 1 |
|  | Yellow | Dongnong303 | 2 |
|  | Red | Gaoyuan 1 | 3 |
|  | Blue | Brigus | 4 |
| Compound leaf size | Minimum | — | 1 |
|  | Minimum to small | — | 2 |
|  | Small | Agassiz | 3 |
|  | Small to medium | — | 4 |
|  | Medium | Kexin4 | 5 |
|  | Medium to large | — | 6 |
|  | Large | Kennebec | 7 |
|  | Large to extremely large | — | 8 |
|  | Extremely large | — | 9 |
| Number of compound leaf lobules | Few | — | 1 |
|  | Few to less | — | 2 |
|  | Less | Kexin19 | 3 |
|  | Less to medium | — | 4 |
|  | Medium | Hua525 | 5 |
|  | Medium to many | — | 6 |
|  | Many | Kexin20 | 7 |
|  | Many to extremely many | — | 8 |
|  | Extremely many | — | 9 |
| Size of parietal lobule | Minimum | — | 1 |
|  | Minimum to small | — | 2 |
|  | Small | — | 3 |
|  | Small to medium | — | 4 |
|  | Medium | — | 5 |
|  | Medium to large | — | 6 |
|  | Large | — | 7 |
|  | Large to extremely large | — | 8 |
|  | Extremely large | — | 9 |
| Plant height | Extremely short | A-6 | 1 |
|  | Very short to short | - | 2 |
|  | Short | Dongnong303 | 3 |
|  | Short to medium | - | 4 |
|  | Medium | Kexin2 | 5 |
|  | Medium to high | - | 6 |
|  | High | Hongtudou | 7 |
|  | High to very high | - | 8 |
|  | Very high | Qingshu9 | 9 |

**Supplemental Table 4 Identification of tuber shape and eye depth**

| ID | Eye depth | Tuber shape | ID | Eye depth | Tuber shape |
| --- | --- | --- | --- | --- | --- |
| ST_1_10 | 5 | 2 | ST_4_11 | 6 | 5 |
| ST_1_12 | 5 | 4 | ST_4_12 | 5 | 3 |
| ST_1_13 | 6 | 1 | ST_4_13 | 6 | 1 |
| ST_1_14 | 2 | 5 | ST_4_14 | 4 | 3 |
| ST_1_15 | 2 | 4 | ST_4_16 | 7 | 4 |
| ST_1_16 | 4 | 4 | ST_4_17 | 5 | 6 |
| ST_1_17 | 5 | 1 | ST_4_18 | 3 | 6 |
| ST_1_18 | 2 | 5 | ST_4_2 | 7 | 2 |
| ST_1_21 | 4 | 6 | ST_4_20 | 4 | 1 |
| ST_1_22 | 3 | 5 | ST_4_21 | 3 | 5 |
| ST_1_23 | 5 | 5 | ST_4_22 | 4 | 3 |
| ST_1_26 | 2 | 4 | ST_4_23 | 6 | 2 |
| ST_1_27 | 4 | 6 | ST_4_25 | 5 | 2 |
| ST_1_28 | 1 | 4 | ST_4_26 | 3 | 5 |
| ST_1_29 | 2 | 4 | ST_4_27 | 3 | 5 |
| ST_1_3 | 3 | 5 | ST_4_28 | 7 | 3 |
| ST_1_31 | 3 | 1 | ST_4_29 | 3 | 3 |
| ST_1_34 | 2 | 4 | ST_4_3 | 4 | 1 |
| ST_1_35 | 2 | 5 | ST_4_30 | 7 | 1 |
| ST_1_36 | 4 | 1 | ST_4_31 | 4 | 2 |
| ST_1_37 | 7 | 3 | ST_4_33 | 4 | 4 |
| ST_1_38 | 5 | 5 | ST_4_36 | 7 | 1 |
| ST_1_39 | 4 | 4 | ST_4_37 | 2 | 4 |
| ST_1_4 | 1 | 4 | ST_4_38 | 2 | 4 |
| ST_1_40 | 3 | 5 | ST_4_39 | 3 | 5 |
| ST_1_41 | 3 | 3 | ST_4_4 | 2 | 4 |
| ST_1_42 | 2 | 4 | ST_4_40 | 5 | 5 |
| ST_1_44 | 3 | 1 | ST_4_41 | 5 | 4 |
| ST_1_45 | 2 | 4 | ST_4_43 | 2 | 4 |
| ST_1_46 | 5 | 4 | ST_4_44 | 6 | 1 |
| ST_1_47 | 4 | 2 | ST_4_45 | 6 | 2 |
| ST_1_48 | 2 | 1 | ST_4_46 | 6 | 2 |
| ST_1_5 | 4 | 4 | ST_4_47 | 4 | 4 |
| ST_1_50 | 6 | 1 | ST_4_48 | 6 | 2 |
| ST_1_53 | 3 | 1 | ST_4_49 | 4 | 1 |
| ST_1_55 | 4 | 5 | ST_4_5 | 3 | 2 |
| ST_1_57 | 5 | 4 | ST_4_51 | 4 | 5 |
| ST_1_58 | 3 | 4 | ST_4_52 | 5 | 2 |
| ST_1_59 | 4 | 3 | ST_4_53 | 3 | 4 |
| ST_1_61 | 4 | 2 | ST_4_54 | 7 | 5 |
| ST_1_62 | 4 | 6 | ST_4_56 | 6 | 5 |
| ST_1_63 | 7 | 4 | ST_4_57 | 5 | 3 |
| ST_1_64 | 4 | 4 | ST_4_58 | 5 | 2 |
| ST_1_65 | 5 | 6 | ST_4_6 | 5 | 3 |
| ST_1_67 | 6 | 3 | ST_4_61 | 4 | 2 |
| ST_1_68 | 4 | 2 | ST_4_62 | 3 | 1 |
| ST_1_69 | 9 | 5 | ST_4_63 | 5 | 5 |
| ST_1_7 | 3 | 4 | ST_4_64 | 7 | 2 |
| ST_1_70 | 3 | 5 | ST_4_65 | 5 | 4 |
| ST_1_71 | 5 | 4 | ST_4_67 | 7 | 1 |
| ST_1_73 | 4 | 4 | ST_4_7 | 4 | 4 |
| ST_1_75 | 3 | 3 | ST_4_72 | 3 | 3 |
| ST_1_76 | 3 | 5 | ST_4_73 | 3 | 4 |
| ST_1_77 | 4 | 2 | ST_4_8 | 6 | 5 |
| ST_1_79 | 6 | 5 | ST_4_83 | 6 | 1 |
| ST_1_8 | 5 | 2 | ST_4_85 | 3 | 5 |
| ST_1_9 | 8 | 6 | ST_4_86 | 3 | 3 |
| ST_CH_3 | 6 | 6 | ST_4_88 | 4 | 3 |
| ST_2_1 | 6 | 4 | ST_4_89 | 5 | 2 |
| ST_2_10 | 3 | 5 | ST_4_9 | 5 | 1 |
| ST_2_14 | - | - | ST_4_91 | 5 | 5 |
| ST_2_15 | 4 | 5 | ST_4_92 | 5 | 4 |
| ST_2_16 | 4 | 5 | ST_4_93 | 6 | 1 |
| ST_2_17 | 7 | 2 | ST_4_94 | 5 | 2 |
| ST_2_18 | 6 | 4 | ST_4_95 | 3 | 3 |
| ST_2_19 | 4 | 6 | ST_4_97 | 6 | 1 |
| ST_2_2 | 5 | 3 | ST_4_98 | 4 | 5 |
| ST_2_20 | 5 | 2 | ST_4_99 | 4 | 6 |
| ST_2_21 | 1 | 4 | ST_5_1 | 7 | 3 |
| ST_2_22 | 5 | 4 | ST_5_10 | 3 | 4 |
| ST_2_24 | 3 | 3 | ST_5_11 | 4 | 3 |
| ST_2_25 | 3 | 4 | ST_5_12 | 4 | 5 |
| ST_2_26 | 3 | 4 | ST_5_13 | 3 | 1 |
| ST_2_27 | 2 | 2 | ST_5_14 | 3 | 5 |
| ST_2_28 | 2 | 4 | ST_5_15 | 3 | 4 |
| ST_2_3 | 9 | 2 | ST_5_16 | 4 | 3 |
| ST_2_30 | 2 | 4 | ST_5_17 | 7 | 3 |
| ST_2_31 | 2 | 4 | ST_5_18 | 5 | 2 |
| ST_2_32 | 2 | 4 | ST_5_19 | 3 | 2 |
| ST_2_33 | 4 | 4 | ST_5_2 | 3 | 3 |
| ST_2_34 | 7 | 1 | ST_5_20 | 4 | 2 |
| ST_2_35 | 6 | 4 | ST_5_21 | 3 | 5 |
| ST_2_39 | 1 | 4 | ST_5_22 | 3 | 5 |
| ST_2_4 | 3 | 4 | ST_5_23 | 3 | 6 |
| ST_2_40 | 3 | 3 | ST_5_24 | 3 | 5 |
| ST_2_41 | 4 | 3 | ST_5_26 | 2 | 4 |
| ST_2_43 | 2 | 4 | ST_5_28 | 2 | 4 |
| ST_2_44 | 3 | 4 | ST_5_29 | 3 | 4 |
| ST_2_45 | 5 | 3 | ST_5_3 | 4 | 4 |
| ST_2_46 | 5 | 5 | ST_5_30 | 4 | 2 |
| ST_2_48 | 7 | 1 | ST_5_31 | 3 | 5 |
| ST_2_49 | 4 | 1 | ST_5_32 | 2 | 2 |
| ST_2_5 | 6 | 5 | ST_5_33 | 3 | 4 |
| ST_2_50 | 3 | 2 | ST_5_34 | 6 | 2 |
| ST_2_52 | 3 | 4 | ST_5_35 | 3 | 4 |
| ST_2_53 | 3 | 6 | ST_5_36 | 2 | 4 |
| ST_2_54 | 8 | 2 | ST_5_37 | 6 | 3 |
| ST_2_55 | 2 | 4 | ST_5_40 | 8 | 1 |
| ST_2_56 | 6 | 2 | ST_5_42 | 4 | 4 |
| ST_2_58 | 2 | 4 | ST_5_43 | 3 | 4 |
| ST_2_62 | 3 | 6 | ST_5_44 | 4 | 4 |
| ST_2_63 | 3 | 4 | ST_5_45 | 4 | 4 |
| ST_2_65 | 6 | 4 | ST_5_47 | 3 | 3 |
| ST_2_68 | 5 | 1 | ST_5_5 | 4 | 3 |
| ST_2_7 | 6 | 2 | ST_5_52 | 4 | 4 |
| ST_2_71 | 5 | 1 | ST_5_53 | 2 | 4 |
| ST_2_72 | 6 | 1 | ST_5_56 | 4 | 4 |
| ST_2_73 | 2 | 4 | ST_5_57 | 3 | 4 |
| ST_2_75 | 4 | 4 | ST_5_58 | 5 | 4 |
| ST_2_76 | 3 | 5 | ST_5_59 | 5 | 3 |
| ST_2_78 | 3 | 4 | ST_5_6 | 3 | 4 |
| ST_2_79 | 2 | 6 | ST_5_60 | 7 | 4 |
| ST_2_8 | 6 | 6 | ST_5_62 | 4 | 1 |
| ST_2_80 | 3 | 6 | ST_5_63 | 3 | 4 |
| ST_2_9 | 4 | 5 | ST_5_65 | 4 | 5 |
| ST_3_1 | 3 | 5 | ST_5_67 | 2 | 4 |
| ST_3_10 | 4 | 4 | ST_5_68 | 3 | 4 |
| ST_3_11 | 5 | 4 | ST_5_69 | 3 | 5 |
| ST_3_12 | 5 | 4 | ST_5_7 | 3 | 4 |
| ST_3_13 | 6 | 2 | ST_5_70 | 6 | 4 |
| ST_3_14 | 4 | 4 | ST_5_72 | 4 | 2 |
| ST_3_15 | 4 | 5 | ST_5_74 | 3 | 3 |
| ST_3_16 | 5 | 1 | ST_5_78 | 5 | 5 |
| ST_3_17 | 5 | 5 | ST_5_79 | 4 | 5 |
| ST_3_18 | 3 | 5 | ST_5_8 | 3 | 2 |
| ST_3_19 | 5 | 5 | ST_5_81 | 5 | 4 |
| ST_3_2 | 4 | 3 | ST_5_82 | 6 | 1 |
| ST_3_20 | 2 | 5 | ST_5_84 | 2 | 5 |
| ST_3_21 | 3 | 5 | ST_5_87 | 5 | 6 |
| ST_3_22 | 2 | 6 | ST_5_88 | 3 | 5 |
| ST_3_23 | 4 | 2 | ST_5_89 | 4 | 2 |
| ST_3_24 | 6 | 5 | ST_5_9 | 1 | 4 |
| ST_3_25 | 4 | 4 | ST_5_91 | 3 | 4 |
| ST_3_27 | 4 | 2 | ST_5_92 | 4 | 3 |
| ST_3_28 | 4 | 5 | ST_6_1 | 2 | 5 |
| ST_3_29 | 4 | 5 | ST_6_11 | 2 | 6 |
| ST_3_3 | 2 | 3 | ST_6_12 | 5 | 4 |
| ST_3_31 | 3 | 4 | ST_6_13 | 2 | 4 |
| ST_3_33 | 3 | 4 | ST_6_14 | 3 | 6 |
| ST_3_35 | 8 | 1 | ST_6_15 | 6 | 3 |
| ST_3_36 | 3 | 6 | ST_6_17 | 3 | 3 |
| ST_3_37 | 5 | 2 | ST_6_18 | 5 | 3 |
| ST_3_38 | 2 | 4 | ST_6_19 | 4 | 4 |
| ST_3_39 | 6 | 4 | ST_6_2 | 5 | 3 |
| ST_3_4 | 2 | 4 | ST_6_20 | 3 | 4 |
| ST_3_40 | 4 | 3 | ST_6_21 | 4 | 4 |
| ST_3_41 | 5 | 2 | ST_6_22 | 4 | 1 |
| ST_3_43 | 5 | 3 | ST_6_23 | 1 | 3 |
| ST_3_47 | 5 | 3 | ST_6_24 | 1 | 4 |
| ST_3_5 | 3 | 5 | ST_6_25 | 2 | 4 |
| ST_3_51 | 4 | 4 | ST_6_26 | 2 | 3 |
| ST_3_52 | 3 | 5 | ST_6_27 | 4 | 5 |
| ST_3_53 | 4 | 3 | ST_6_28 | 4 | 1 |
| ST_3_59 | 4 | 4 | ST_6_3 | 3 | 3 |
| ST_3_6 | 4 | 2 | ST_6_32 | 2 | 2 |
| ST_3_60 | 4 | 6 | ST_6_33 | 2 | 4 |
| ST_3_61 | 3 | 4 | ST_6_34 | 3 | 4 |
| ST_3_63 | 7 | 4 | ST_6_36 | 6 | 6 |
| ST_3_64 | 2 | 5 | ST_6_38 | 8 | 3 |
| ST_3_65 | 2 | 4 | ST_6_39 | 2 | 4 |
| ST_3_66 | 2 | 4 | ST_6_4 | 2 | 3 |
| ST_3_68 | 7 | 2 | ST_6_40 | 7 | 2 |
| ST_3_7 | 3 | 5 | ST_6_43 | 2 | 4 |
| ST_3_70 | 3 | 2 | ST_6_45 | 3 | 1 |
| ST_3_74 | 4 | 3 | ST_6_48 | 2 | 4 |
| ST_3_76 | 6 | 2 | ST_6_49 | 4 | 4 |
| ST_3_77 | 3 | 4 | ST_6_50 | 5 | 2 |
| ST_3_8 | 6 | 2 | ST_6_51 | 2 | 3 |
| ST_3_83 | 3 | 2 | ST_6_52 | 2 | 4 |
| ST_3_87 | 3 | 3 | ST_6_53 | 2 | 4 |
| ST_3_88 | 5 | 4 | ST_6_54 | 3 | 4 |
| ST_3_89 | 5 | 5 | ST_6_57 | 3 | 4 |
| ST_3_9 | 4 | 5 | ST_6_58 | 5 | 2 |
| ST_3_90 | 3 | 5 | ST_6_63 | 1 | 4 |
| ST_3_91 | 4 | 5 | ST_6_67 | 4 | 4 |
| ST_3_92 | 2 | 5 | ST_6_7 | 3 | 4 |
| ST_3_93 | 4 | 4 | ST_6_8 | 3 | 5 |
| ST_3_94 | 5 | 4 | ST_6_81 | 5 | 2 |
| ST_3_95 | 7 | 4 | ST_6_83 | 2 | 4 |
| ST_3_96 | 4 | 5 | ST_6_84 | 4 | 2 |
| ST_3_97 | 3 | 5 | ST_6_85 | 3 | 4 |
| ST_3_98 | 6 | 2 | ST_6_86 | 4 | 3 |
| ST_3_99 | 5 | 5 | ST_6_88 | 5 | 5 |
| ST_4_1 | 5 | 1 | ST_6_89 | 4 | 1 |
| ST_4_10 | 5 | 6 | ST_6_9 | 2 | 4 |

**Supplemental Table 5** Overview of SNPs and insertion deletion sites

| Types | EXON | INTRON | UTR_3_PRIME | UTR_5_PRIME | INTERGENIC |
| --- | --- | --- | --- | --- | --- |
| Snps | 181,309 | 539,122 | 31,949 | 17,388 | 3,761,148 |
| Indels | 7,713 | 78,504 | 6,183 | 3,057 | 354,435 |

**Supplemental Table 6** Significant SNP sites in the potato tuber shape

| Chr. | Position | P | Chr. | Position | P |
| --- | --- | --- | --- | --- | --- |
| Chr01A1 | 34440295 | 7.10E-05 | Chr02A1 | 28398953 | 5.99E-05 |
| Chr01A1 | 34451395 | 5.14E-06 | Chr02A1 | 28399270 | 1.14E-05 |
| Chr01A1 | 34451510 | 9.62E-06 | Chr02A1 | 28401210 | 2.25E-05 |
| Chr01A1 | 34453239 | 5.96E-05 | Chr02A1 | 28404564 | 6.71E-06 |
| Chr01A1 | 34458899 | 4.00E-06 | Chr02A1 | 28406078 | 5.02E-05 |
| Chr01A1 | 34463060 | 1.41E-05 | Chr02A1 | 28490297 | 8.61E-05 |
| Chr01A1 | 34464256 | 8.41E-05 | Chr02A1 | 28490312 | 9.05E-05 |
| Chr01A1 | 34464361 | 2.40E-05 | Chr02A1 | 28490315 | 9.05E-05 |
| Chr01A1 | 34466909 | 8.68E-06 | Chr02A1 | 28520985 | 6.00E-05 |
| Chr01A1 | 34468187 | 6.98E-05 | Chr02A1 | 28522330 | 7.59E-05 |
| Chr01A1 | 34468332 | 6.06E-05 | Chr02A1 | 28522339 | 9.56E-05 |
| Chr01A1 | 34468354 | 5.66E-05 | Chr02A1 | 28543657 | 1.89E-05 |
| Chr01A1 | 34468389 | 6.06E-05 | Chr02A1 | 28543714 | 7.70E-05 |
| Chr01A1 | 34472838 | 3.26E-05 | Chr02A1 | 3135487 | 3.91E-05 |
| Chr01A1 | 34477187 | 6.14E-05 | Chr02A1 | 5119450 | 5.38E-05 |
| Chr01A1 | 34477568 | 4.08E-05 | Chr02A1 | 5284509 | 4.77E-05 |
| Chr01A1 | 34481897 | 1.88E-05 | Chr02A1 | 5284565 | 1.79E-05 |
| Chr01A1 | 34482559 | 3.13E-05 | Chr02A1 | 5471722 | 5.27E-05 |
| Chr01A1 | 34484034 | 3.35E-06 | Chr02A1 | 5471732 | 5.27E-05 |
| Chr01A1 | 34484958 | 6.10E-06 | Chr02A1 | 7795199 | 3.86E-05 |
| Chr01A1 | 34485577 | 7.63E-05 | Chr02A1 | 7795202 | 3.86E-05 |
| Chr01A1 | 34503329 | 7.31E-06 | Chr02A1 | 8645047 | 8.10E-05 |
| Chr01A1 | 34504451 | 3.44E-05 | Chr02A1 | 8645049 | 8.10E-05 |
| Chr01A1 | 34521558 | 6.37E-05 | Chr02A2 | 27780967 | 9.34E-05 |
| Chr01A1 | 34669362 | 3.40E-05 | Chr02A2 | 40752536 | 1.29E-05 |
| Chr01A1 | 34673312 | 7.14E-05 | Chr02A2 | 43245606 | 8.74E-05 |
| Chr01A1 | 34673396 | 8.76E-05 | Chr02A2 | 43245657 | 6.45E-05 |
| Chr01A1 | 34679259 | 5.44E-05 | Chr02A2 | 44727625 | 9.22E-05 |
| Chr01A1 | 34685805 | 8.21E-05 | Chr02A3 | 11130095 | 2.98E-05 |
| Chr01A1 | 34685807 | 6.76E-05 | Chr02A3 | 11583799 | 1.09E-05 |
| Chr01A1 | 34688418 | 4.08E-05 | Chr02A3 | 11583807 | 3.42E-05 |
| Chr01A1 | 34688531 | 9.95E-05 | Chr02A3 | 11744836 | 5.50E-06 |
| Chr01A1 | 34689241 | 8.98E-05 | Chr02A3 | 13896562 | 1.58E-05 |
| Chr01A1 | 34690044 | 3.85E-06 | Chr02A3 | 13900454 | 2.37E-05 |
| Chr01A1 | 34690478 | 6.82E-05 | Chr02A3 | 13900532 | 3.13E-05 |
| Chr01A1 | 34690645 | 6.14E-05 | Chr02A3 | 29732447 | 7.45E-05 |
| Chr01A1 | 34690910 | 4.98E-05 | Chr02A3 | 30190240 | 2.35E-05 |
| Chr01A1 | 34691399 | 6.24E-05 | Chr02A4 | 22223570 | 1.18E-05 |
| Chr01A1 | 34692064 | 5.00E-05 | Chr03A2 | 12452193 | 1.85E-05 |
| Chr01A1 | 34694588 | 9.18E-05 | Chr03A3 | 22031375 | 8.25E-05 |
| Chr01A1 | 34695281 | 2.06E-05 | Chr03A3 | 22320217 | 3.20E-06 |
| Chr01A1 | 34695339 | 6.30E-05 | Chr03A3 | 23083342 | 1.35E-05 |
| Chr01A1 | 34700965 | 5.57E-05 | Chr03A3 | 23083346 | 2.95E-05 |
| Chr01A1 | 34720389 | 1.47E-05 | Chr03A3 | 23268921 | 1.35E-05 |
| Chr01A1 | 34721657 | 9.67E-05 | Chr03A3 | 23268941 | 3.01E-05 |
| Chr01A1 | 34738398 | 5.45E-05 | Chr03A3 | 23330618 | 6.79E-05 |
| Chr01A1 | 34740914 | 9.21E-06 | Chr03A3 | 28256061 | 4.71E-05 |
| Chr01A1 | 34790018 | 6.17E-05 | Chr03A3 | 28709169 | 2.35E-05 |
| Chr01A1 | 34793147 | 2.34E-05 | Chr03A3 | 51010594 | 2.99E-05 |
| Chr01A1 | 34796741 | 5.82E-05 | Chr03A3 | 51010896 | 6.64E-05 |
| Chr01A1 | 35246668 | 5.72E-05 | Chr03A3 | 51011803 | 2.56E-05 |
| Chr01A2 | 63230182 | 8.57E-05 | Chr03A3 | 51011809 | 4.11E-05 |
| Chr01A4 | 19701306 | 9.29E-05 | Chr03A3 | 51011907 | 6.54E-05 |
| Chr02A1 | 17250622 | 8.82E-05 | Chr03A3 | 51012036 | 1.73E-05 |
| Chr02A1 | 28347697 | 3.86E-05 | Chr03A3 | 51012466 | 6.83E-05 |
| Chr02A1 | 28359920 | 9.41E-06 | Chr03A3 | 51012468 | 5.92E-05 |
| Chr02A1 | 28360022 | 6.55E-06 | Chr03A3 | 51024946 | 9.12E-05 |
| Chr02A1 | 28360028 | 2.64E-06 | Chr03A3 | 51028436 | 7.38E-05 |
| Chr02A1 | 28361526 | 2.79E-05 | Chr03A3 | 51030302 | 6.24E-05 |
| Chr02A1 | 28362877 | 8.59E-06 | Chr03A3 | 5996412 | 4.88E-05 |
| Chr02A1 | 28363008 | 9.96E-05 | Chr03A3 | 5996420 | 2.30E-05 |
| Chr02A1 | 28364890 | 2.06E-05 | Chr03A3 | 5996435 | 8.37E-05 |
| Chr02A1 | 28364902 | 2.06E-05 | Chr05A2 | 17323857 | 5.55E-05 |
| Chr02A1 | 28364973 | 3.91E-05 | Chr05A2 | 29137229 | 7.28E-05 |
| Chr02A1 | 28381405 | 2.61E-05 | Chr05A2 | 31897978 | 9.33E-05 |
| Chr02A1 | 28381527 | 1.21E-05 | Chr05A3 | 19760960 | 8.41E-05 |
| Chr02A1 | 28381647 | 3.89E-06 | Chr05A3 | 23218948 | 9.15E-05 |
| Chr02A1 | 28381654 | 3.89E-06 | Chr05A3 | 33372386 | 8.21E-05 |
| Chr02A1 | 28382620 | 5.21E-05 | Chr06A2 | 45941388 | 6.84E-05 |
| Chr02A1 | 28387041 | 3.57E-05 | Chr07A2 | 25940514 | 1.39E-05 |
| Chr02A1 | 28397921 | 6.39E-05 | Chr09A3 | 46955370 | 8.72E-05 |
| Chr02A1 | 28398045 | 8.09E-05 | Chr11A3 | 2037364 | 6.99E-06 |
| Chr02A1 | 28398699 | 2.28E-05 | Chr11A4 | 25587702 | 2.85E-05 |

**Supplemental Table 7** Significant loci on Chr01A1

| Position (bp) | 34451395 | 34458899 | 34466909 | 34484034 | 34484958 | 34503329 | Observations |
| --- | --- | --- | --- | --- | --- | --- | --- |
| Alleles | T/C | T/A | A/G | T/G | T/C | C/A | - |
| ST_1_48 | C | - | G | G | C | A | 1 |
| ST_1_50 | C | A | G | G | C | A | 1 |
| ST_1_53 | C | A | G | G | C | A | 1 |
| ST_2_35 | C | T | G | G | C | A | 1 |
| ST_2_49 | C | T | G | G | C | A | 1 |
| ST_2_5 | C | A | G | G | C | - | 1 |
| ST_2_7 | C | A | G | G | C | A | 1 |
| ST_3_17 | C | A | G | G | C | A | 1 |
| ST_3_36 | C | A | G | G | C | - | 1 |
| ST_4_10 | C | A | G | G | C | A | 1 |
| ST_4_14 | C | A | G | G | C | A | 1 |
| ST_4_30 | - | A | G | G | C | - | 1 |
| ST_4_31 | C | T | G | G | C | A | 1 |
| ST_4_37 | C | T | G | G | C | A | 1 |
| ST_4_85 | C | A | G | G | C | A | 1 |
| ST_1_62 | - | - | - | - | - | - | 6 |
| ST_1_65 | - | - | - | - | - | - | 6 |
| ST_2_1 | - | - | - | - | - | - | 6 |
| ST_2_2 | - | - | - | - | - | - | 6 |
| ST_2_54 | - | - | - | - | - | - | 6 |
| ST_2_63 | - | - | - | - | - | - | 6 |
| ST_2_80 | - | - | - | - | - | - | 6 |
| ST_2_9 | C | - | - | G | C | A | 6 |
| ST_3_23 | - | - | - | - | - | - | 6 |
| ST_3_37 | - | - | - | - | - | - | 6 |
| ST_4_11 | - | - | - | T | T | C | 6 |
| ST_4_18 | - | - | - | - | - | - | 6 |
| ST_4_2 | - | - | - | - | T | - | 6 |
| ST_5_1 | T | T | A | T | T | - | 6 |
| ST_6_15 | - | - | - | - | - | - | 6 |

**Supplemental Table 8** Significant loci on Chr02A1

| Position (bp) | 28359920 | 28360022 | 28360028 | 28362877 | 28381647 | 28381654 | Observations |
| --- | --- | --- | --- | --- | --- | --- | --- |
| Alleles | T/C | T/C | C/T | G/T | A/C | T/C | - |
| ST_1_48 | C | C | T | T | C | C | 1 |
| ST_1_50 | C | C | T | T | C | C | 1 |
| ST_1_53 | C | C | T | T | C | C | 1 |
| ST_2_35 | C | C | T | T | C | C | 1 |
| ST_2_49 | C | C | T | T | C | C | 1 |
| ST_2_5 | C | C | T | T | C | C | 1 |
| ST_2_7 | C | C | T | T | C | C | 1 |
| ST_3_17 | C | C | T | T | C | C | 1 |
| ST_3_36 | C | C | T | T | C | C | 1 |
| ST_4_10 | C | C | T | T | C | C | 1 |
| ST_4_14 | C | C | T | T | C | C | 1 |
| ST_4_30 | C | C | T | T | C | C | 1 |
| ST_4_31 | C | C | T | T | C | C | 1 |
| ST_4_37 | C | C | T | T | C | C | 1 |
| ST_4_85 | C | C | T | T | C | C | 1 |
| ST_1_62 | - | - | - | - | - | - | 6 |
| ST_1_65 | - | - | - | - | - | - | 6 |
| ST_2_1 | T | T | C | G | A | T | 6 |
| ST_2_2 | - | - | - | - | - | - | 6 |
| ST_2_54 | - | - | - | - | - | - | 6 |
| ST_2_63 | - | - | - | - | - | - | 6 |
| ST_2_80 | - | - | - | - | - | - | 6 |
| ST_2_9 | - | - | - | - | - | - | 6 |
| ST_3_23 | - | - | - | T | C | C | 6 |
| ST_3_37 | - | - | - | - | - | - | 6 |
| ST_4_11 | T | T | C | G | A | T | 6 |
| ST_4_18 | T | T | C | G | A | T | 6 |
| ST_4_2 | - | - | - | - | - | - | 6 |
| ST_5_1 | - | - | - | - | - | - | 6 |
| ST_6_15 | - | - | - | - | - | - | 6 |

**Supplemental Table 9** Candidate genes for tuber shape association analysis

| Gene ID | Chr. | Start | End | Description |
| --- | --- | --- | --- | --- |
| Soltu.Q9.Chr01A10001728.g | Chr01A1 | 34407582 | 34413055 | Serine threonine-protein phosphatase |
| Soltu.Q9.Chr01A10001730.g | Chr01A1 | 34578881 | 34580946 | PIF1-like helicase |
| Soltu.Q9.Chr02A10004900.g | Chr02A1 | 17196944 | 17197984 | beta-hydroxysteroid-dehydrogenase decarboxylase |
| Soltu.Q9.Chr02A10005623.g | Chr02A1 | 28355588 | 28358622 | PLATZ transcription factor |
| Soltu.Q9.Chr02A10005624.g | Chr02A1 | 28373137 | 28382116 | UTP--glucose-1-phosphate uridylyltransferase |
| Soltu.Q9.Chr02A10005625.g | Chr02A1 | 28387314 | 28398490 | FAR1 DNA-binding domain |
| Soltu.Q9.Chr02A10005636.g | Chr02A1 | 28546884 | 28556633 | Receptor kinase galacturonan-binding |
| Soltu.Q9.Chr02A20005458.g | Chr02A2 | 27756525 | 27758715 | Oxidoreductase |
| Soltu.Q9.Chr02A20005459.g | Chr02A2 | 27767465 | 27768949 | Oxidoreductase |
| Soltu.Q9.Chr02A20006701.g | Chr02A2 | 40719331 | 40728073 | NPH3 family |
| Soltu.Q9.Chr02A20006702.g | Chr02A2 | 40730480 | 40740518 | Serine/Threonine protein kinases |
| Soltu.Q9.Chr02A20006703.g | Chr02A2 | 40738684 | 40740549 | Zinc-binding |
| Soltu.Q9.Chr02A20006712.g | Chr02A2 | 40792769 | 40796291 | Sigma factor PP2C-like phosphatases |
| Soltu.Q9.Chr02A20006948.g | Chr02A2 | 43209577 | 43211817 | PMR5 N terminal Domain |
| Soltu.Q9.Chr02A20006950.g | Chr02A2 | 43234772 | 43237908 | Glycosyl transferase family 21 |
| Soltu.Q9.Chr02A20006951.g | Chr02A2 | 43251712 | 43254409 | Proline dehydrogenase |
| Soltu.Q9.Chr02A20006954.g | Chr02A2 | 43275712 | 43276800 | S-adenosylmethionine decarboxylase |
| Soltu.Q9.Chr02A20006956.g | Chr02A2 | 43284668 | 43285485 | Stress-induced protein KIN2-like |
| Soltu.Q9.Chr02A20007109.g | Chr02A2 | 44683418 | 44690160 | Glycosyltransferase 1 |
| Soltu.Q9.Chr02A20007112.g | Chr02A2 | 44717573 | 44718991 | Myb-like DNA-binding domain |
| Soltu.Q9.Chr02A30004458.g | Chr02A3 | 11127982 | 11128509 | ATPase activity |
| Soltu.Q9.Chr02A30004468.g | Chr02A3 | 11591636 | 11592223 | ATPase activity |
| Soltu.Q9.Chr02A30005628.g | Chr02A3 | 29688724 | 29702381 | Starch synthase |
| Soltu.Q9.Chr02A40005854.g | Chr02A4 | 22202175 | 22218997 | SIT4 phosphatase-associated protein |
| Soltu.Q9.Chr03A20008230.g | Chr03A2 | 12397763 | 12399311 | Protein serine/threonine kinase activity |
| Soltu.Q9.Chr03A30007389.g | Chr03A3 | 6012307 | 6013236 | NmrA-like family |
| Soltu.Q9.Chr03A30007391.g | Chr03A3 | 6027179 | 6031668 | Methylesterase 10-like |
| Soltu.Q9.Chr03A30007393.g | Chr03A3 | 6039296 | 6039631 | Belongs to the cytochrome P450 family |
| Soltu.Q9.Chr03A30007394.g | Chr03A3 | 6048731 | 6049829 | Belongs to the cytochrome P450 family |
| Soltu.Q9.Chr03A30007876.g | Chr03A3 | 22292649 | 22293679 | Myb-like DNA-binding domain |
| Soltu.Q9.Chr03A30008038.g | Chr03A3 | 28222106 | 28222720 | ATPase activity |
| Soltu.Q9.Chr03A30009213.g | Chr03A3 | 50953654 | 50958177 | Alpha/beta hydrolase fold |
| Soltu.Q9.Chr03A30009216.g | Chr03A3 | 50981238 | 50991398 | FES |
| Soltu.Q9.Chr03A30009217.g | Chr03A3 | 51000965 | 51002593 | Zinc-finger of the FCS-type, C2-C2 |
| Soltu.Q9.Chr03A30009218.g | Chr03A3 | 51023444 | 51032329 | DNA recombination |
| Soltu.Q9.Chr03A30009219.g | Chr03A3 | 51053440 | 51057155 | Transcription factor |
| Soltu.Q9.Chr03A30009220.g | Chr03A3 | 51057947 | 51059504 | Late embryogenesis abundant protein |
| Soltu.Q9.Chr03A30009221.g | Chr03A3 | 51062889 | 51063736 | GDSL lipolytic enzyme family |
| Soltu.Q9.Chr03A30009222.g | Chr03A3 | 51072028 | 51079319 | Phosphoinositide |
| Soltu.Q9.Chr03A30009223.g | Chr03A3 | 51082215 | 51092574 | CBF/Mak21 family |
| Soltu.Q9.Chr05A30014606.g | Chr05A3 | 33334405 | 33339703 | Zinc finger protein |
| Soltu.Q9.Chr06A20016862.g | Chr06A2 | 45902974 | 45914986 | AIG1 family |
| Soltu.Q9.Chr06A20016869.g | Chr06A2 | 45953561 | 45957561 | Carbon-nitrogen hydrolase |
| Soltu.Q9.Chr06A20016870.g | Chr06A2 | 45958689 | 45963971 | Alpha/beta hydrolase family |
| Soltu.Q9.Chr06A20016873.g | Chr06A2 | 45982614 | 45988567 | Agamous-like MADS-box protein AGL11 |
| Soltu.Q9.Chr09A30025673.g | Chr09A3 | 46948390 | 46955937 | 3-isopropylmalate dehydratase |
| Soltu.Q9.Chr09A30025674.g | Chr09A3 | 46958022 | 46961163 | Aux IAA proteins |
| Soltu.Q9.Chr09A30025675.g | Chr09A3 | 46970184 | 46972181 | Protein kinase C conserved region 2 |
| Soltu.Q9.Chr11A30027991.g | Chr11A3 | 1996780 | 2004544 | Glycosyl hydrolase family 9 |
| Soltu.Q9.Chr11A30027992.g | Chr11A3 | 2004636 | 2012494 | Beta-hexosaminidase |

**Supplemental Table 10** Potato tuber shape related candidate transcription factors

| Gene ID | Family | Best hit in *A. thaliana* | Description for the best hit |
| --- | --- | --- | --- |
| Soltu.Q9.Chr05A30014606.g | CO-like | AT5G48250.1 | B-box type zinc finger protein |
| Soltu.Q9.Chr03A30009219.g | ERF | AT1G53910.1 | Related to AP2 12 |
| Soltu.Q9.Chr02A10005625.g | FAR1 | AT4G15090.1 | FAR1 family protein |
| Soltu.Q9.Chr06A20016873.g | MADS | AT4G09960.2 | MIKC_MADS family protein |
| Soltu.Q9.Chr02A20007112.g | MYB | AT3G47600.1 | MYB domain protein 94 |
| Soltu.Q9.Chr03A30007876.g | MYB | AT3G28470.1 | MYB family protein |

**Supplemental Table 11** Significant SNP loci in the potato tuber bud eye depth

| Chr. | Position | P | Chr. | Position | P |
| --- | --- | --- | --- | --- | --- |
| Chr01A1 | 15154972 | 6.06E-05 | Chr05A2 | 50087357 | 8.93E-06 |
| Chr01A1 | 35222018 | 9.30E-05 | Chr05A2 | 50087467 | 1.89E-05 |
| Chr01A1 | 36641717 | 8.93E-05 | Chr05A2 | 50143917 | 3.36E-05 |
| Chr01A1 | 55542 | 4.17E-05 | Chr05A2 | 50385093 | 5.25E-05 |
| Chr01A1 | 7803795 | 9.88E-05 | Chr05A2 | 50385385 | 8.55E-05 |
| Chr01A1 | 8000599 | 4.16E-05 | Chr05A3 | 30829883 | 2.69E-05 |
| Chr01A4 | 21617302 | 5.44E-05 | Chr06A2 | 25851419 | 6.66E-05 |
| Chr01A4 | 33261563 | 5.45E-05 | Chr06A2 | 25895980 | 1.98E-06 |
| Chr01A4 | 9302556 | 9.14E-05 | Chr06A2 | 25906311 | 9.12E-05 |
| Chr02A1 | 5050148 | 5.46E-05 | Chr06A2 | 26381384 | 4.87E-05 |
| Chr02A1 | 9654827 | 2.12E-05 | Chr06A2 | 26386612 | 7.65E-05 |
| Chr02A3 | 28029756 | 4.95E-05 | Chr07A4 | 41322162 | 7.63E-05 |
| Chr02A4 | 15402087 | 9.93E-05 | Chr07A4 | 41607432 | 4.10E-05 |
| Chr02A4 | 2799534 | 6.32E-05 | Chr07A4 | 6045791 | 7.39E-05 |
| Chr03A3 | 51012791 | 2.85E-05 | Chr08A3 | 641844 | 4.80E-06 |
| Chr03A3 | 51012793 | 9.73E-05 | Chr08A4 | 22879650 | 1.06E-05 |
| Chr04A1 | 38127599 | 5.98E-05 | Chr08A4 | 22879701 | 5.23E-05 |
| Chr04A1 | 4618882 | 4.20E-06 | Chr08A4 | 22879708 | 4.16E-05 |
| Chr04A2 | 46139696 | 3.12E-05 | Chr09A1 | 13703884 | 4.14E-05 |
| Chr05A1 | 29782177 | 3.67E-05 | Chr09A2 | 10047929 | 5.23E-05 |
| Chr05A1 | 30332382 | 8.20E-06 | Chr11A2 | 1879367 | 5.70E-05 |
| Chr05A1 | 9553297 | 7.12E-05 | Chr11A2 | 1972040 | 1.75E-05 |
| Chr05A2 | 50031597 | 6.19E-05 | Chr11A3 | 14130683 | 9.59E-05 |
| Chr05A2 | 50087233 | 2.07E-05 | Chr11A3 | 31586573 | 7.70E-05 |
| Chr05A2 | 50087238 | 4.68E-05 | Chr11A3 | 31746272 | 9.22E-05 |
| Chr05A2 | 50087243 | 4.83E-05 | Chr11A3 | 5160071 | 2.71E-06 |
| Chr05A2 | 50087245 | 4.83E-05 |  |  |  |

**Supplemental Table 12** Significant loci on Chr05A2

| Position (bp) | 50031597 | 50087233 | 50087238 | 50087243 | 50087245 | 50087357 | 50087467 | 50143917 | 50385093 | 50385385 | Observations |
| --- | --- | --- | --- | --- | --- | --- | --- | --- | --- | --- | --- |
| alleles | G/A | C/A | A/G | A/G | A/G | C/G | A/T | T/C | T/C | C/G | - |
| ST_1_28 | A | A | G | G | G | G | T | C | C | G | 1 |
| ST_1_4 | A | A | G | G | G | G | T | C | C | G | 1 |
| ST_6_24 | A | A | G | G | G | G | T | C | C | G | 1 |
| ST_6_25 | A | A | G | G | G | G | T | C | C | G | 1 |
| ST_6_67 | A | A | G | G | G | G | T | C | C | G | 1 |
| ST_1_29 | A | A | G | G | G | G | T | C | C | G | 2 |
| ST_1_34 | A | A | G | G | G | G | T | C | C | G | 2 |
| ST_1_35 | A | A | G | G | G | G | T | C | C | G | 2 |
| ST_1_48 | A | A | G | G | G | G | T | C | C | G | 2 |
| ST_2_44 | A | A | G | G | G | G | T | C | C | G | 2 |
| ST_3_39 | A | A | G | G | G | G | T | C | C | G | 2 |
| ST_3_40 | A | A | G | G | G | G | T | C | C | G | 2 |
| ST_3_66 | A | A | G | G | G | G | T | C | C | G | 2 |
| ST_3_68 | A | A | G | G | G | G | T | C | C | G | 2 |
| ST_4_38 | A | A | G | G | G | G | T | C | C | G | 2 |
| ST_1_63 | G | C | A | A | A | C | A | T | - | C | 7 |
| ST_2_18 | G | C | A | A | A | C | A | T | T | C | 7 |
| ST_2_35 | - | - | - | - | - | - | - | - | - | - | 7 |
| ST_2_49 | - | - | - | - | - | - | - | T | - | - | 7 |
| ST_3_64 | - | - | - | - | - | - | - | - | - | - | 7 |
| ST_3_7 | G | C | A | A | A | C | A | T | T | C | 7 |
| ST_3_96 | - | - | - | - | - | - | - | - | - | - | 7 |
| ST_4_17 | - | - | - | - | - | - | - | - | - | - | 7 |
| ST_4_29 | - | - | - | - | - | - | - | - | - | - | 7 |
| ST_4_56 | G | C | A | A | A | C | A | - | T | C | 7 |
| ST_4_7 | - | - | - | - | - | - | - | - | - | - | 7 |
| ST_5_10 | - | - | - | - | - | - | - | - | - | - | 7 |
| ST_5_18 | G | C | A | A | A | C | A | T | T | C | 7 |
| ST_5_62 | G | C | A | A | A | C | A | T | T | C | 7 |
| ST_6_43 | - | - | - | - | - | - | - | - | - | - | 7 |
| ST_3_36 | G | C | A | A | A | C | A | - | T | C | 8 |
| ST_5_42 | - | - | - | - | - | - | - | - | - | - | 8 |
| ST_2_30 | G | - | - | - | - | - | - | - | - | - | 9 |

**Supplemental Table 13** Candidate genes for Tuber bud eye depth association analysis

| Gene ID | Chr. | Start | End | Description |
| --- | --- | --- | --- | --- |
| Soltu.Q9.Chr01A10000712.g | Chr01A1 | 7744172 | 7744699 | Leucine-rich repeat receptor-like protein kinase |
| Soltu.Q9.Chr01A10000713.g | Chr01A1 | 7770909 | 7774168 | Peroxidase |
| Soltu.Q9.Chr01A10000717.g | Chr01A1 | 7807451 | 7808138 | B3 DNA binding domain |
| Soltu.Q9.Chr01A10000730.g | Chr01A1 | 7977834 | 7979933 | Belongs to the peroxidase family |
| Soltu.Q9.Chr01A10000734.g | Chr01A1 | 8004857 | 8009758 | UDP-D-apiose UDP-D-xylose synthase |
| Soltu.Q9.Chr01A10001150.g | Chr01A1 | 15212760 | 15217905 | UAA transporter family |
| Soltu.Q9.Chr01A40000751.g | Chr01A4 | 9321077 | 9325521 | Glycosyl hydrolase family |
| Soltu.Q9.Chr01A40000756.g | Chr01A4 | 9357564 | 9370909 | Protein phosphatase 2A homologues |
| Soltu.Q9.Chr02A30005489.g | Chr02A3 | 28025781 | 28030601 | Vesicle transport protein GOT1B-like |
| Soltu.Q9.Chr02A40005343.g | Chr02A4 | 15416283 | 15423747 | Ubiquitin family |
| Soltu.Q9.Chr03A30009213.g | Chr03A3 | 50953654 | 50958177 | Alpha/beta hydrolase fold |
| Soltu.Q9.Chr03A30009216.g | Chr03A3 | 50981238 | 50991398 | FES |
| Soltu.Q9.Chr03A30009217.g | Chr03A3 | 51000965 | 51002593 | Zinc-finger of the FCS-type, C2-C2 |
| Soltu.Q9.Chr03A30009219.g | Chr03A3 | 51053440 | 51057155 | Transcription factor |
| Soltu.Q9.Chr03A30009220.g | Chr03A3 | 51057947 | 51059504 | Late embryogenesis abundant protein |
| Soltu.Q9.Chr03A30009221.g | Chr03A3 | 51062889 | 51063736 | GDSL lipolytic enzyme family |
| Soltu.Q9.Chr03A30009222.g | Chr03A3 | 51072028 | 51079319 | Phosphoinositide |
| Soltu.Q9.Chr04A10010032.g | Chr04A1 | 4568385 | 4569475 | Endoglucanase |
| Soltu.Q9.Chr04A10010038.g | Chr04A1 | 4605174 | 4610255 | E3 ubiquitin-protein ligase |
| Soltu.Q9.Chr04A10010039.g | Chr04A1 | 4622890 | 4623684 | Transcription factor |
| Soltu.Q9.Chr04A10010045.g | Chr04A1 | 4643545 | 4646417 | Mitosis protein DIM1 |
| Soltu.Q9.Chr04A20012331.g | Chr04A2 | 46095891 | 46103017 | Permease family |
| Soltu.Q9.Chr04A20012332.g | Chr04A2 | 46114073 | 46123916 | SERine Proteinase INhibitors |
| Soltu.Q9.Chr04A20012335.g | Chr04A2 | 46153087 | 46168483 | POT family |
| Soltu.Q9.Chr04A20012336.g | Chr04A2 | 46168278 | 46176650 | Signal peptidase, peptidase S26 |
| Soltu.Q9.Chr04A20012337.g | Chr04A2 | 46189480 | 46194738 | Transmembrane amino acid transporter protein |
| Soltu.Q9.Chr05A10013282.g | Chr05A1 | 9500032 | 9502353 | Alliin lyase |
| Soltu.Q9.Chr05A10013286.g | Chr05A1 | 9595452 | 9597407 | palmitoyl-（protein） hydrolase activity |
| Soltu.Q9.Chr05A10014387.g | Chr05A1 | 29777468 | 29781282 | Zinc finger, C2H2 type |
| Soltu.Q9.Chr05A10014389.g | Chr05A1 | 29794126 | 29798444 | Belongs to the group II decarboxylase family |
| Soltu.Q9.Chr05A10014390.g | Chr05A1 | 29802977 | 29810455 | UTP--glucose-1-phosphate uridylyltransferase |
| Soltu.Q9.Chr05A10014393.g | Chr05A1 | 29826171 | 29826699 | E3 ubiquitin-protein ligase |
| Soltu.Q9.Chr05A10014430.g | Chr05A1 | 30281905 | 30284354 | GATA transcription factor |
| Soltu.Q9.Chr05A10014432.g | Chr05A1 | 30300640 | 30303006 | Myb-like DNA-binding domain |
| Soltu.Q9.Chr05A10014438.g | Chr05A1 | 30364605 | 30369230 | Glycosyltransferase like family 2 |
| Soltu.Q9.Chr05A20014796.g | Chr05A2 | 49968389 | 49983094 | Aspartic proteinase-like protein 2 isoform X1 |
| Soltu.Q9.Chr05A20014797.g | Chr05A2 | 50015084 | 50018380 | Ras-related protein |
| Soltu.Q9.Chr05A20014806.g | Chr05A2 | 50109912 | 50114551 | Ulp1 protease |
| Soltu.Q9.Chr05A20014807.g | Chr05A2 | 50116227 | 50118589 | Protein FAR1-RELATED SEQUENCE |
| Soltu.Q9.Chr05A20014824.g | Chr05A2 | 50340044 | 50347387 | Belongs to the Nudix hydrolase family |
| Soltu.Q9.Chr05A20014825.g | Chr05A2 | 50349614 | 50359183 | SAM-binding methyltransferase |
| Soltu.Q9.Chr06A20015844.g | Chr06A2 | 25800471 | 25800771 | CYPs |
| Soltu.Q9.Chr06A20015845.g | Chr06A2 | 25800932 | 25801556 | CYPs |
| Soltu.Q9.Chr06A20015846.g | Chr06A2 | 25805350 | 25810629 | CYPs |
| Soltu.Q9.Chr07A40017936.g | Chr07A4 | 5990452 | 5991293 | Myb-like DNA-binding domain |
| Soltu.Q9.Chr07A40017937.g | Chr07A4 | 6021753 | 6023621 | Aux IAA proteins |
| Soltu.Q9.Chr07A40017938.g | Chr07A4 | 6028377 | 6029177 | Ulp1 protease |
| Soltu.Q9.Chr07A40017939.g | Chr07A4 | 6039236 | 6040357 | Glycosyltransferase family 17 |
| Soltu.Q9.Chr07A40017944.g | Chr07A4 | 6094589 | 6115818 | WD40 repeats |
| Soltu.Q9.Chr07A40019498.g | Chr07A4 | 41277813 | 41280373 | Indole-3-acetic acid-amido synthetase |
| Soltu.Q9.Chr07A40019502.g | Chr07A4 | 41321308 | 41325137 | Serine threonine-protein kinase |
| Soltu.Q9.Chr07A40019528.g | Chr07A4 | 41627844 | 41628840 | MYB domain class transcription factor |
| Soltu.Q9.Chr07A40019529.g | Chr07A4 | 41657381 | 41658521 | MYB domain class transcription factor |
| Soltu.Q9.Chr08A30021648.g | Chr08A3 | 583201 | 586539 | Alpha-IPM synthase homocitrate synthase |
| Soltu.Q9.Chr08A30021650.g | Chr08A3 | 631593 | 633612 | Alpha-IPM synthase homocitrate synthase |
| Soltu.Q9.Chr08A40020993.g | Chr08A4 | 22854774 | 22856614 | Oxidoreductase activity |
| Soltu.Q9.Chr09A10022605.g | Chr09A1 | 13742159 | 13744417 | Serine threonine-protein kinase |
| Soltu.Q9.Chr09A20022935.g | Chr09A2 | 9997420 | 9998064 | Abscisic acid receptor |
| Soltu.Q9.Chr11A20026834.g | Chr11A2 | 1823075 | 1826733 | Ribosomal protein L23 |
| Soltu.Q9.Chr11A20026838.g | Chr11A2 | 1846682 | 1849870 | GDSL lipolytic enzyme |
| Soltu.Q9.Chr11A20026839.g | Chr11A2 | 1854611 | 1859469 | Glycosyl hydrolase 17 |
| Soltu.Q9.Chr11A20026843.g | Chr11A2 | 1917423 | 1919142 | Glycosyl hydrolase 17 |
| Soltu.Q9.Chr11A20026844.g | Chr11A2 | 1919185 | 1919569 | Glycosyl hydrolase 17 |
| Soltu.Q9.Chr11A30028273.g | Chr11A3 | 5119811 | 5122132 | tRNA nucleotidyltransferase |
| Soltu.Q9.Chr11A30028274.g | Chr11A3 | 5126800 | 5128376 | tRNA nucleotidyltransferase |
| Soltu.Q9.Chr11A30028275.g | Chr11A3 | 5129734 | 5135838 | tRNA nucleotidyltransferase |
| Soltu.Q9.Chr11A30028278.g | Chr11A3 | 5168940 | 5169530 | Plant invertase/pectin methylesterase inhibitor |
| Soltu.Q9.Chr11A30029409.g | Chr11A3 | 31588813 | 31591334 | MIP aquaporin |
| Soltu.Q9.Chr11A30029414.g | Chr11A3 | 31624772 | 31629220 | Glutaredoxin |
| Soltu.Q9.Chr11A30029415.g | Chr11A3 | 31630106 | 31635384 | Myb-like DNA-binding domain |
| Soltu.Q9.Chr11A30029420.g | Chr11A3 | 31695213 | 31699013 | Mads box protein |
| Soltu.Q9.Chr11A30029421.g | Chr11A3 | 31728417 | 31735504 | OTU-like cysteine protease |
| Soltu.Q9.Chr11A30029422.g | Chr11A3 | 31754658 | 31759587 | HSP60 |
| Soltu.Q9.Chr11A30029424.g | Chr11A3 | 31799184 | 31808520 | OTU-like cysteine protease |

**Supplemental Table 14** Potato tuber eye depth related candidate transcription factors

| TF ID | Family | Best hit in *A. thaliana* | Description for the best hit |
| --- | --- | --- | --- |
| Soltu.Q9.Chr01A10000717.g | B3 | AT3G18990.1 | B3 family protein |
| Soltu.Q9.Chr03A30009219.g | ERF | AT1G53910.1 | related to AP2 12 |
| Soltu.Q9.Chr04A10010039.g | TCP | AT5G23280.1 | TCP family protein |
| Soltu.Q9.Chr05A10014387.g | C2H2 | AT2G01940.1 | C2H2-like zinc finger protein |
| Soltu.Q9.Chr05A10014430.g | GATA | AT3G06740.1 | GATA transcription factor 15 |
| Soltu.Q9.Chr05A10014432.g | MYB | AT5G49330.1 | MYB domain protein 111 |
| Soltu.Q9.Chr07A40017936.g | MYB | AT5G52600.1 | MYB domain protein 82 |
| Soltu.Q9.Chr07A40019528.g | MYB | AT2G31180.1 | MYB domain protein 14 |
| Soltu.Q9.Chr07A40019529.g | MYB | AT2G31180.1 | MYB domain protein 14 |
| Soltu.Q9.Chr11A30029415.g | MYB_related | AT1G72650.2 | TRF-like 6 |
| Soltu.Q9.Chr11A30029420.g | MADS | AT5G60440.1 | AGAMOUS-like 62 |
